# The Mitochondrial Guardian α‐Amyrin Mitigates Alzheimer's Disease Pathology via Modulation of the DLK‐SARM1‐ULK1 Axis

**DOI:** 10.1002/advs.202512374

**Published:** 2026-01-22

**Authors:** Shu‐Qin Cao, Juan Ignacio Jiménez‐Loygorri, Yunguang Qiu, You Jung Kang, Khanh Van Do, Annabel E. Smith, Junjie Huang, Jun‐ping Pan, Lipeng Mao, Ang Li, Hangge Yang, Yahyah Aman, Maria Jose Donate Lagartos, Sofie Hindkjær Lautrup, Anbin Chen, Kristina Xiao Liang, Hailong Zhang, Juan Yi, Xurui Jin, Tin Cho Cheung, Olympia Apokotou, Florentia Papastefanaki, Rebecca Matsas, William A. McEwan, Feixiong Cheng, Hansang Cho, Guobing Chen, Huanxing Su, Konstantinos Palikaras, Oscar Junhong Luo, Qian Hua Zhao, Ding Ding, Cornelia van Duijn, Nektarios Tavernarakis, Patricia Boya, Tewin Tencomnao, Evandro F. Fang

**Affiliations:** ^1^ Department of Clinical Molecular Biology University of Oslo and Akershus University Hospital Lørenskog Norway; ^2^ Department of Clinical Chemistry Faculty of Allied Health Sciences Chulalongkorn University Pathum Wan Bangkok Thailand; ^3^ Department of Cellular and Molecular Biology Margarita Salas Center For Biological Research Spanish National Research Council Madrid Madrid Spain; ^4^ Cleveland Clinic Genome Center Cleveland Clinic Research Cleveland Clinic Cleveland Ohio USA; ^5^ Department of Genomic Sciences and Systems Biology Cleveland Clinic Research, Cleveland Clinic Cleveland Ohio USA; ^6^ Institute of Quantum Biophysics Sungkyunkwan University Suwon Gyeonggi Republic of Korea; ^7^ Department of Biophysics Sungkyunkwan University Suwon Gyeonggi Republic of Korea; ^8^ Department of Intelligent Precision Healthcare Convergence Sungkyunkwan University Suwon Gyeonggi Republic of Korea; ^9^ UK Dementia Research Institute at the University of Cambridge Cambridge UK; ^10^ Department of Clinical Neurosciences University of Cambridge Cambridge UK; ^11^ MindRank AI Ltd. Hangzhou Zhejiang P. R. China; ^12^ Department of Microbiology and Immunology School of Medicine Institute of Geriatric Immunology School of Medicine Jinan University Guangzhou P. R. China; ^13^ Department of Systems Biomedical Sciences School of Medicine Jinan University Guangzhou P. R. China; ^14^ Guangdong‐Hong Kong‐Macau Great Bay Area Geroscience Joint Laboratory Guangzhou P. R. China; ^15^ State Key Laboratory of Mechanism and Quality of Chinese Medicine Institute of Chinese Medical Sciences University of Macau Macao P. R. China; ^16^ School of Basic Medical Sciences Lanzhou University Lanzhou Gansu P. R. China; ^17^ Department of Clinical Medicine (K1) University of Bergen Bergen Norway; ^18^ Department of Neurosurgery Xinhua Hospital Affiliated to Shanghai Jiaotong University School of Medicine Shanghai P. R. China; ^19^ Hong Kong Longevity Science Laboratory Limited Hong Kong Science Park Hong Kong SAR P. R. China; ^20^ Department of Neurobiology Hellenic Pasteur Institute Athens Greece; ^21^ Department of Molecular Medicine, Cleveland Clinic Lerner College of Medicine, Case Western Reserve University, Cleveland, Ohio, USA Cleveland Clinic Lerner College of Medicine, Case Western Reserve University Cleveland Ohio USA; ^22^ Department of Physiology Medical School National and Kapodistrian University of Athens Athens Greece; ^23^ Institute of Neurology Huashan Hospital Fudan University Shanghai P. R. China; ^24^ National Center for Neurological Disorders Huashan Hospital Fudan University Shanghai P. R. China; ^25^ National Clinical Research Center for Aging and Medicine Huashan Hospital Fudan University Shanghai P. R. China; ^26^ Nuffield Department of Population Health The University of Oxford Oxford UK; ^27^ Institute of Molecular Biology and Biotechnology Foundation For Research and Technology‐Hellas Heraklion Crete Greece; ^28^ Department of Basic Sciences Faculty of Medicine University of Crete Heraklion Crete Greece; ^29^ Departament of Neuroscience and Movement Science Section Medicine University of Fribourg Fribourg Switzerland; ^30^ Center of Excellence on Natural Products for Neuroprotection and Anti‐ageing (Neur‐Age NatChula) Chulalongkorn University Pathum Wan Bangkok Thailand; ^31^ The Norwegian Centre on Healthy Ageing (NO‐Age) and the Norwegian National anti‐Alzheimer's Disease (NO‐AD) Networks Oslo Norway

**Keywords:** α‐Amyrin, artificial intelligence, cognitive function, dual leucine zipper kinase (DLK), mitochondrial function

## Abstract

High consumption of colorful fruits and vegetables correlates with low dementia risk, but the exact molecules and the underlying biological mechanisms governing their bioactive profiles are largely unknown. Using a 10‐year observational cohort study coupled with an AI‐driven systems pharmacology platform, we identified a natural triterpenoid compound found in colorful fruits and vegetables, α‐Amyrin (αA), as a therapeutic candidate for Alzheimer's disease (AD). The efficacy of αA in treating the symptoms of AD, such as Tau tangles, damaged mitochondria, and memory loss, was examined using cross‐species models; αA retained memory in AD‐like animal models while also strongly inhibiting Tau pathology, especially p‐Tau217, in a cellular ‘Tau seeding’ system and in Tau[P301S] mice, followed by validation using a human 3D microfluidic system. At molecular level, αA is a robust mitochondrial regulator, enhancing mitochondrial stress resilience and activation of mitophagy. Mechanistically, αA inhibits dual leucine zipper kinase (DLK), leading to the inhibition of DLK‐Sterile Alpha and TIR Motif Containing 1 (SARM1)‐dependent neurodegeneration; this inhibition frees unc‐51 Like Autophagy Activating Kinase 1 (ULK1) from the ULK1‐SARM1 complex, allowing it to participate in autophagy/mitophagy. αA also shows strong translational potential with a 10.1 h half‐life and the ability to cross the blood‐brain barrier. Our results indicate that αA may act as a mitochondrial guardian against AD via modulating the DLK‐SARM1‐ULK1‐autophagy/mitophagy axis while further preclinical and clinical studies are warranted.

## Graphical abstract

Dietary habits play a key role in chronic diseases, and higher annual consumption of fruit and vegetable may lower risk of dementia. Artificial intelligence predicts the lipid‐like compound α‐Amyrin (αA) from plants with edible peels as a drug candidate against Alzheimer's disease. The authors suggest that αA promotes cellular mitophagy (mitochondrial self‐clearance) and secures energy production, which then slows down memory loss.

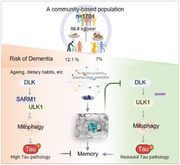

## 1 Introduction

The global healthcare system faces immense challenges and tremendous financial and social burdens due to the rapidly ageing global population; ageing itself is associated with a rising prevalence of chronic disorders, including dementia, cardiovascular disease, and diabetes [1, 2]. Unlike other irreversible risk factors such as genetics and chronological aging, dietary habits represent a modifiable risk factor for chronic diseases [3]. Recent studies suggest that a diet high in colorful fruits and vegetables may reduce the risk of aging‐associated cognitive decline. For example, studies from both the German DZNE‐ cohort [4] and the U.S. MAP cohort [5] indicated that a Mediterranean‐like diet may reduce the risk of cognitive decline and Alzheimer's disease (AD) pathology. As well, a U.S.‐based randomized controlled trial highlighted that dietary flavanols restored hippocampal‐dependent memory in older adults [6]. However, our understanding of the identities of related biomedical molecules in these fruits and vegetables and the mechanisms by which they affect brain health are still largely unknown.

AD is the most common dementia, currently affecting over 35 million individuals worldwide, and is expected to affect up to 100 million individuals by 2050 [7]. AD imposes a progressive cognitive and emotional burden on affected individuals, their families, and caregivers who often face psychological distress, emotional strain, and reduced life quality as the disease advances. Two disease‐defining pathophysiological features of AD include extracellular Aβ plaques (high intracellular Aβ_1‐42_ is also toxic) and intracellular hyperphosphorylated Tau (p‐Tau)‐induced neurofibrillary tangles (NFTs), contributing to cognitive decline [8, 9, 10]. Tau protein, encoded by the *microtubule‐associated protein Tau* (*MAPT*) gene, plays important roles in microtubule assembly and stabilization, neurite outgrowth, and axonal transport [11, 12]. Hyperphosphorylation and aggregation of Tau in neuronal and/or glial cells are associated with diseases called tauopathies, which include AD and other neurodegenerative diseases [13]. Accumulation of p‐Tau217 (pTau that is phosphorylated at position 217) disrupts synaptic integrity and impairs the hippocampal and cortical circuitry essential for memory consolidation. Recent studies postulate that plasma p‐Tau217 holds potential as an early predictive marker of AD progression [14, 15, 16, 17]. Investigation into whether targeting Tau pathology may offer dual benefits, ameliorating both neurobiological and neuropsychiatric manifestations of the disease, is warranted.

Mitochondria are essential organelles responsible for production of the energy required for proper neuronal function; in AD, these organelles become impaired and unproductive [18, 19]. Mitophagy, a sub‐type of macroautophagy that selectively removes and recycles damaged or superfluous mitochondria [9], plays a critical role in maintaining mitochondrial homeostasis and quality, as well as ensuring efficient production of adenosine triphosphate (ATP) [9, 20]. Our previous studies found that impaired mitophagy contributes to AD progression (including higher p‐Tau [20] and memory loss), and that mitophagy‐stimulating natural molecules show promise as therapeutic agents for AD [21, 22, 23]. Thus, pharmacological strategies that improve mitochondrial homeostasis and upregulate mitophagy might be potential solutions for tauopathies.

The Shanghai Aging Study is a well‐documented and quality‐controlled longitudinal cohort providing a unique source for studying AD risks in a representative sample of the Asian population [16, 24]. We examined a 10‐year longitudinal nutritional data set from this study and found that high fruit and vegetable consumption correlates with low plasma p‐Tau217 (a biomarker for AD) and a reduced risk of dementia in community‐dwelling older adults. We identified α‐Amyrin (αA), a pentacyclic triterpenol available in fruits like passion fruit, as a potent mitophagy inducer with anti‐AD potential; additionally, we characterized its pharmacokinetics, protein‐binding target in cells, and its ability to alleviate cognitive deficits via modified mitochondria function.

## 2 Results

### 2.1 There Is an Inverse Correlation Between Fruit and Vegetable Consumption and Dementia Risk/Plasma p‐Tau217 Levels

**FIGURE 1:**
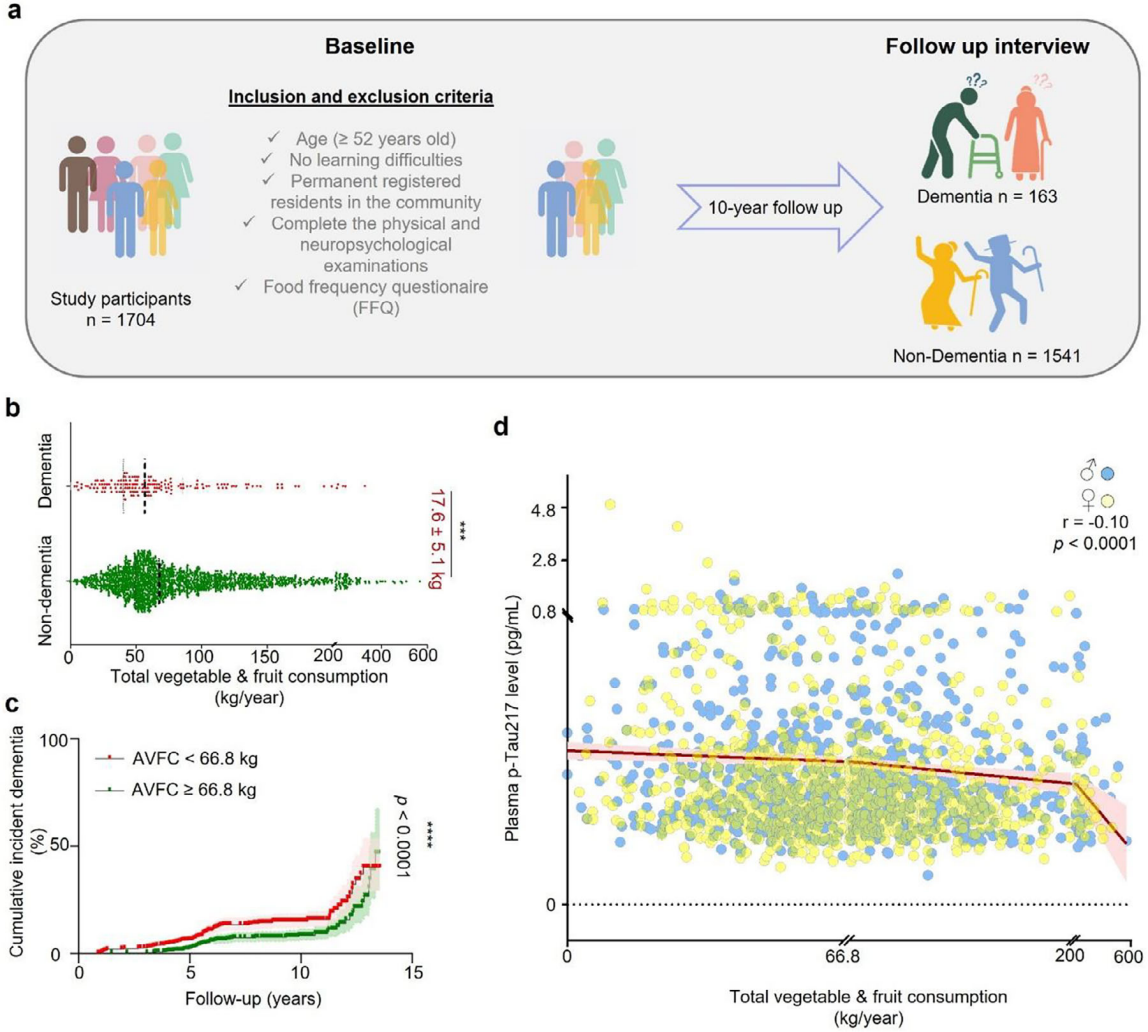
Correlation between annual vegetable/fruit consumption, dementia and plasma p‐Tau 217 at baseline. (a) The inclusion and exclusion criteria for the study participants are shown. (b) Annual fruit and vegetable consumption (AFVC) is shown for participants with and without dementia. (c) Cumulative incident dementia is shown for participants with high AFVC (≥ 66.8 kg/year) or low AFVC (< 66.8 kg/year). (d) AFVC and plasma p‐Tau217 at baseline for male and female study participants. Data are presented as mean ± S.E.M. Statistical analyses used were as follows: unpaired t test (b), the Kaplan–Meier curve with Log‐rank test (c). and Pearson's correlation coefficient r test (d). Tests were set at a significance level of 0.05, ***p < 0.001, ****p < 0.0001.

A cohort of 1704 dementia‐free participants from the Shanghai Aging Study (baseline mean age = 69.2 years) were followed prospectively from 2010–2011 to 2023. At baseline, participants were administered a food frequency questionnaire (FFQ) and their plasma p‐Tau217 was quantified [16]. The median length of follow‐up was 5.7 years (range, 0.9 – 13.5). Cognitive function was assessed at baseline and in follow‐up years [16]. After 10 years, 1541 (90.4%) participants remained dementia‐free, and 163 (9.6%) participants demonstrated cognitive impairment consistent with dementia (Figure 1a). Information provided on the FFQ at baseline was used to calculate annual fruit and vegetable consumption (AFVC) for each participant. AFVC of non‐dementia participants (mean = 87.6 kg/year) was significantly higher than AFVC for participants with dementia (mean = 70.1 kg/year) (Figure 1b). We also compared dementia risk in participants with high AFVC (≥ 66.8 kg/year) to dementia risk in participants with low AFVC (<66.8 kg/year) and found that low and high median AFVC corresponds to 12.1% and 7.0% risk of dementia, respectively (Figure 1c). In addition, AFVC is inversely correlated (r = ‐0.01, *p* < 0.0001) with baseline concentration of plasma p‐Tau217 (0.39 [0.28] pg/mL) (Figure 1d).

### 2.2 AI‐driven Systems Pharmacology Predicts Anti‐AD Potential of Naturally Occurring Compounds from Fruit Extracts

**FIGURE 2:**
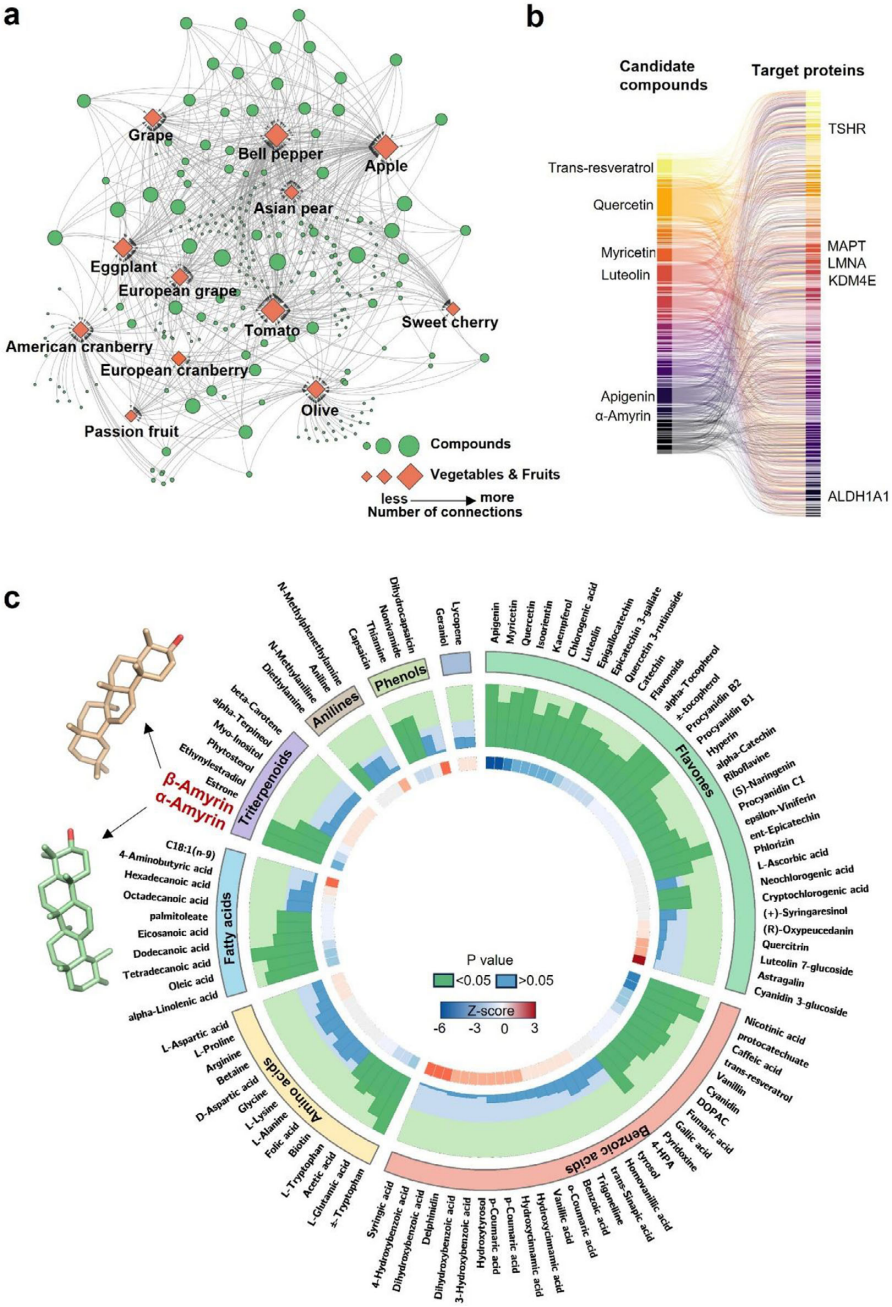
Computational search for fruit extract‐derived neuroprotective compounds and top anti‐AD candidates. (a) Candidate compounds (*n* = 240) from 11 plant species were collected from the food composition database FooDB (www.foodb.ca) and from literature [21]. Diamonds represent source fruits, and circles represent candidate compounds. The size of the node corresponds to the number of compounds/fruits connected. The panel a is created by Cytoscape 3.9.1. (b) Sankey diagram representing 317 predicted protein‐ligand pairs involving 145 unique AD‐seed proteins and 102 fruit‐derived compounds validated using InChIKey (https://www.inchi‐trust.org). The top five targets, five compounds, and α‐Amyrin are labelled. (c) Circos plot showing prioritized candidate compounds based on proximity network analysis. The outer circle represents chemical categories of fruit‐derived compounds organized by cluster matched with 145 AD seed proteins using a database of 351 445 compound‐protein interactions. The inner circle displays *p* values calculated by proximity network analysis for each compound. Two colors are used to distinguish significant associations between compounds and AD. The green indicates *p* < 0.05, while blue indicates *p* > 0.05. The innermost circle represents the Z‐score calculated by proximity network analysis for each compound. A more negative z‐score indicates a high‐confidence in predicted associations between the compound and AD.

Edible fruits and vegetables represent the main source of bioactive and most likely low toxicity compounds. For example, extracts of *Passiflora edulis* (passion fruit) stimulate mitophagy, improve cognitive function, and normalize neurological defects in a *Caenorhabditis elegans* model of AD [21]. To investigate the therapeutic potential of bioactive compounds in colorful fruits and vegetables, we used a food composition database (www.foodb.ca) and relevant literature sources and focused on 240 candidate compounds detected in tomato, apple, bell pepper, Asian pear, grape, eggplant, European grape, American cranberry, passion fruit, olive, and sweet cherry (Figure 2a). Using an AI‐driven systems pharmacology approach, we built a network of candidate compounds using Cytoscape 3.9.1 and presented compound‐protein interactions using network proximity analysis and InChIKey (see Methods for details). Here, the most common hits were α‐amyrin (αA), α‐tocopherol, and phosphorus, compounds that are abundant in 10 out of 11 of the fruit species examined (Figure 2a). Next, we retrieved and analyzed published bioactive target proteins from the reference databases ChEMBL and BindingDB (see methods). Of 240 candidate compounds, we identified 102 compounds with established biological potency and 317 corresponding target proteins (Figure 2b). The top 5 target proteins were thyrotropin receptor (encoded by gene *TSHR*), Tau (*MAPT*), Lamin A (*LMNA*), Lysine demethylase 4E (*KDM4E*), and Aldehyde dehydrogenase 1 A1 (*ALDH1A1*); the top 5 compounds were trans‐resveratrol, quercetin, myricetin, luteolin, and apigenin (Figure 2b). Hierarchical clustering of 102 compounds identified 10 clusters based on chemical structure (Figure S1a), including flavones (cluster 1, *n* = 33), benzoic acids (cluster 2, *n* = 27), triterpenoids (cluster 3, *n* = 8), amino acids (cluster 4, *n* = 10), and fatty acids (cluster 5, *n* = 10). Network proximity analysis and an in‐house‐generated protein‐protein interaction network [25, 26] were used to rank and prioritize the candidate compounds by z score (lower z score, higher predicted relevance to AD) relative to known AD‐relevant proteins (Figure 2c) [27]. These analyses pointed to alpha‐amyrin (αA), a pentacyclic triterpenol, as a lead compound with high predicted anti‐AD activity (z‐score = −4.198). Other top candidate compounds were apigenin (z‐score = −5.403), myricetin (z‐score = −5.281), quercetin (z‐score = −4.559), nicotinic acid (z‐score = −4.533), protocatechuate (z‐score = −4.512), isoorientin (z‐score = −4.467), kaempferol (z‐score = −4.388), chlorogenic acid (z‐score = −4.112), and luteolin (z‐score = −4.07) (Table S1). Most of these compounds are commercially available; phenolic acids and flavonoids are common dietary supplements. αA is present in common fruits and vegetables, including American cranberry, Asian pear, bell pepper, eggplant, European cranberry, grape, olive, passion fruit, sweet cherry, and tomato, and so we selected αA (z‐score = −4.198) and its isomer β‐Amyrin (βA) (z score = −2.852, *p* < 0.001) for further study (Figure 2c).

### 2.3 α‐Amyrin Inhibits Tau Aggregation and Promotes Tau Degradation

**FIGURE 3:**
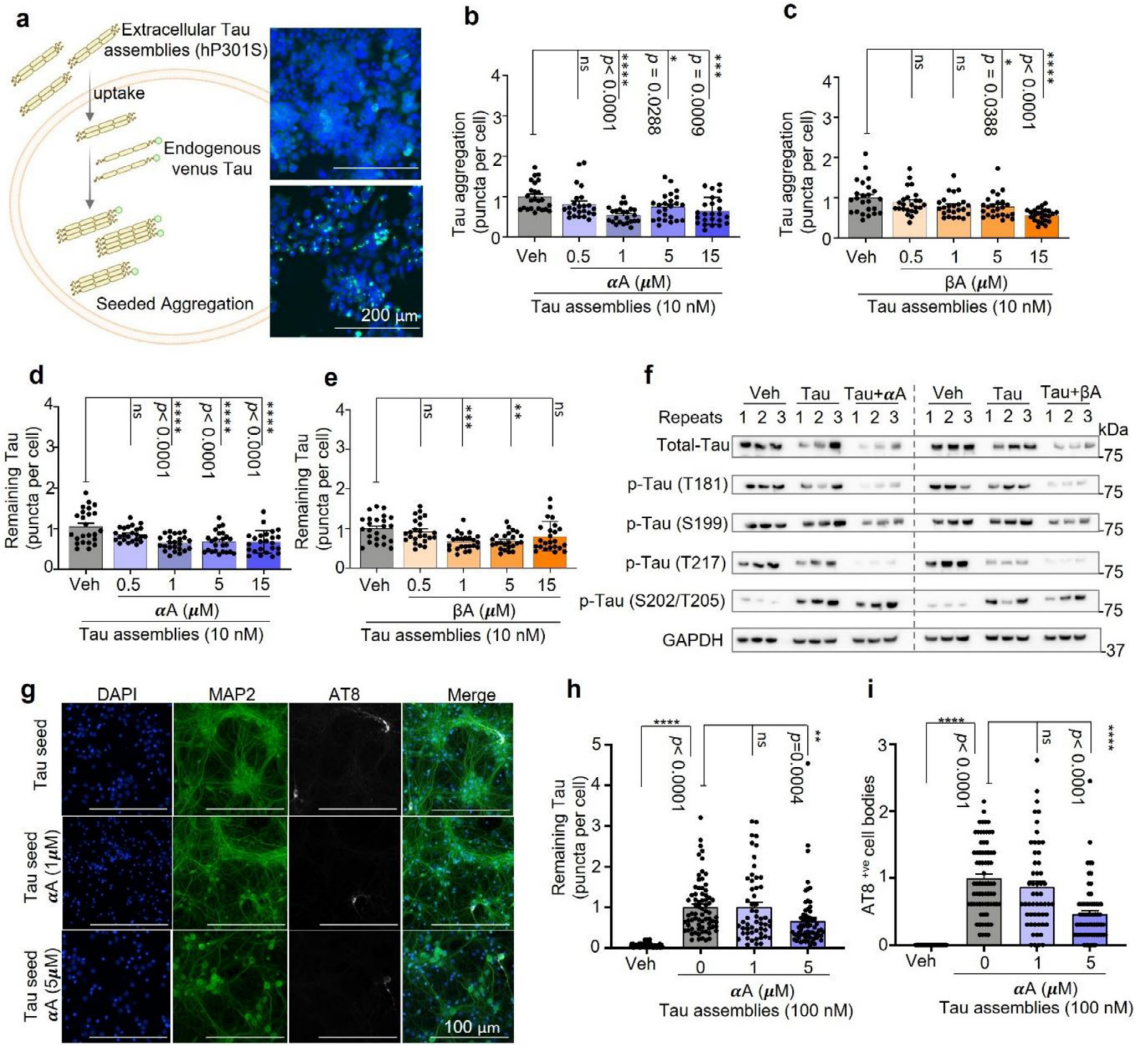
Effect of α‐Amyrin on aggregation and phosphorylation of Tau P301S in HEK293 cells and murine primary neurons. (a) Schematic diagram showing how exogenous recombinant heparin‐assembled P301S Tau aggregates (Tau assemblies) trigger aggregation of endogenous Venus‐tagged P301S Tau in HEK293 cells (left). Representative images (right) of HEK293 cells expressing endogenous P301S Tau‐Venus in the presence or absence of Tau seeds (10 nm, 72 h). Green indicates Tau aggregates, and blue represents nuclear. Scale bar= 200 µm. (b) Cells were exposed to vehicle or 0.5, 1, 5, or 15 µm αA for 72 h after ‘Tau assemblies’ seeded immediately, and Tau aggregates quantified. (c) As in (b), except cells were exposed to 0.5, 1, 5, or 15 µm βA. (d) Cells were exposed to vehicle or 0.5, 1, 5, or 15 µm αA for 48 h after 48 h ‘Tau assemblies’ seeding; degradation of Tau aggregates was quantified. (e) As in (d), except cells were exposed to 0.5, 1, 5, or 15 µm βA. (f) Western blot to determine total Tau and Thr181, Ser199, Thr217, Ser202/Thr205 phosphorylated Tau variants in the absence (vehicle) or presence of αA or βA (1 µm, 48 h) as indicated. (g) Representative immunofluorescence images of hTau:P301S murine primary neurons cultured for 7 days in the absence or presence of 100 nm Tau assemblies without (vehicle) or with αA (1, 5 µm, overnight). Green indicates Tau aggregates, and blue represents nuclear. Scale bar = 100 µmm. (h) Quantification of AT8‐staining foci (puncta, p‐Tau Ser202/Thr205) in hTau:P301S‐expressing murine primary neurons pre‐treated without (vehicle) or with αA (1, 5 µm) prior to addition of 100 nm Tau seeds for 7 days. Data were normalized to vehicle control. (i) Quantification of AT8‐positive hTau:P301S‐expressing murine primary neurons pre‐treated without (vehicle) or with αA (1, 5 µm) prior to addition of 100 nm Tau assemblies for 7 days. All values were normalized to vehicle control. Unless specified elsewhere, data are mean ± S.E.M. Statistical analyses used were as follows: one‐way ANOVA followed by Dunnett's multiple comparisons test (b‐e, h, i). All panels: *n.s*., not significant, *p < 0.05, **p < 0.01, ***p < 0.001, ****p < 0.0001.

A high level of p‐Tau tends to cause Tau aggregation and formation of insoluble deposits which are a hallmark of AD; agents that limit p‐Tau production or promote its degradation are thought to have therapeutic potential [23, 28]. To investigate the capacity of αA or βA to antagonize p‐Tau‐related pathology, a well‐established and validated in vitro ‘Tau seeding’ system was used [29]. Briefly, exogenous Tau assemblies were added to human HEK293 cells stably expressing the 0N4R isoform of hTau P301S with a C‐terminal fluorescent Venus tag (HEK293T Venus) to induce the endogenous mutant Tau to form Tau aggregates which were then detected as fluorescent intracellular puncta (Figure 3a, Figure S1b). After safe dose selection (0.125 to 30 µM) for αA or βA (Figure S1c,d), Tau seeding was performed for two detections to investigate how αA or βA (0.5, 1, 5, or 15 µm) affected Tau pathology. We monitored αA/βA's effect on Tau assemblies‐induced Tau aggregate formation and found that αA reduced Tau aggregation by 17.9%, 45.4%, 25.0%, and 35.3%, at 0.5, 1, 5, and 15 µm respectively (Figure 3b), while βA reduced Tau aggregation by 11.3%, 21.2%, 22.2%, and 43.5%, respectively (Figure 3c) at the same concentrations. We also looked at whether αA/βA were able to alleviate the degradation of preformed Tau aggregates and found that αA reduced aggregated Tau by 19.5%, 40.1%, 38.1% and 38.2% at 0.5, 1, 5, and 15 µm respectively, after 48 h (Figure 3d) while, βA reduced aggregated Tau by 7.5%, 32.8%, 31.1%, and 19.6% respectively after 48 h (Figure 3e); neither were able to reduce aggregation after only 24 h (Figure S1e,f). Tau aggregate formation and phosphorylated Tau accumulation are inextricably linked. Therefore, we assessed the expression level of Tau protein in 4 specific phospho‐variants of Tau (Thr181, Ser199, Thr217, and Ser202/Thr205) in the absence and presence of αA or βA (1 µM, 48 h) using Western blot analysis. Interestingly, Tau assemblies had no effect on Tau phospho‐variants Thr181, Ser199 or Thr217, but increased phosphorylation at Ser202/Thr205 6.5‐fold (Figure 3f; and quantification in Figure S1g,h). αA reduced phosphorylation at Thr181 and Thr217 by 49.5% and 62.4%, respectively, but had no effect on Tau phosphorylation at Ser202/Thr205 (Figure 3f; Figure S1g). Similarly, βA reduced phosphorylation of Thr181 by 54.3%, but had no significant effect on phosphorylation in other Tau residues (Figure 3f; Figure S1h). Since αA showed greater efficacy against hTau‐related pathology than βA, we moved forward with testing of αA and checked how αA affects Tau assembly‐induced Tau aggregate formation using primary neurons from hTau:P301S transgenic mice [30]. The primary neurons were exposed to Tau assemblies (100 nM) with or without αA (1, 5 µm) pretreatment. Cultures were then stained for total Tau (anti‐MAP2) or AT8, a monoclonal antibody specific for p‐Tau Ser202/Thr205. We found that 5 µM αA reduced p‐Tau aggregation by 33.8% (Figure 3g,h) and p‐Tau Ser202/Thr205 phosphorylation by 53.4% (Figure 3g,i) relative to vehicle control. While varied doses of αA were required in these Tau seeding systems, αA was still able to reduce phosphorylation and aggregation of Tau, and so the translational potential of αA was investigated further.

### 2.4 α‐Amyrin Exhibits a Favorable Pharmacokinetic Profile and Crosses the Blood‐Brain Barrier

**FIGURE 4:**
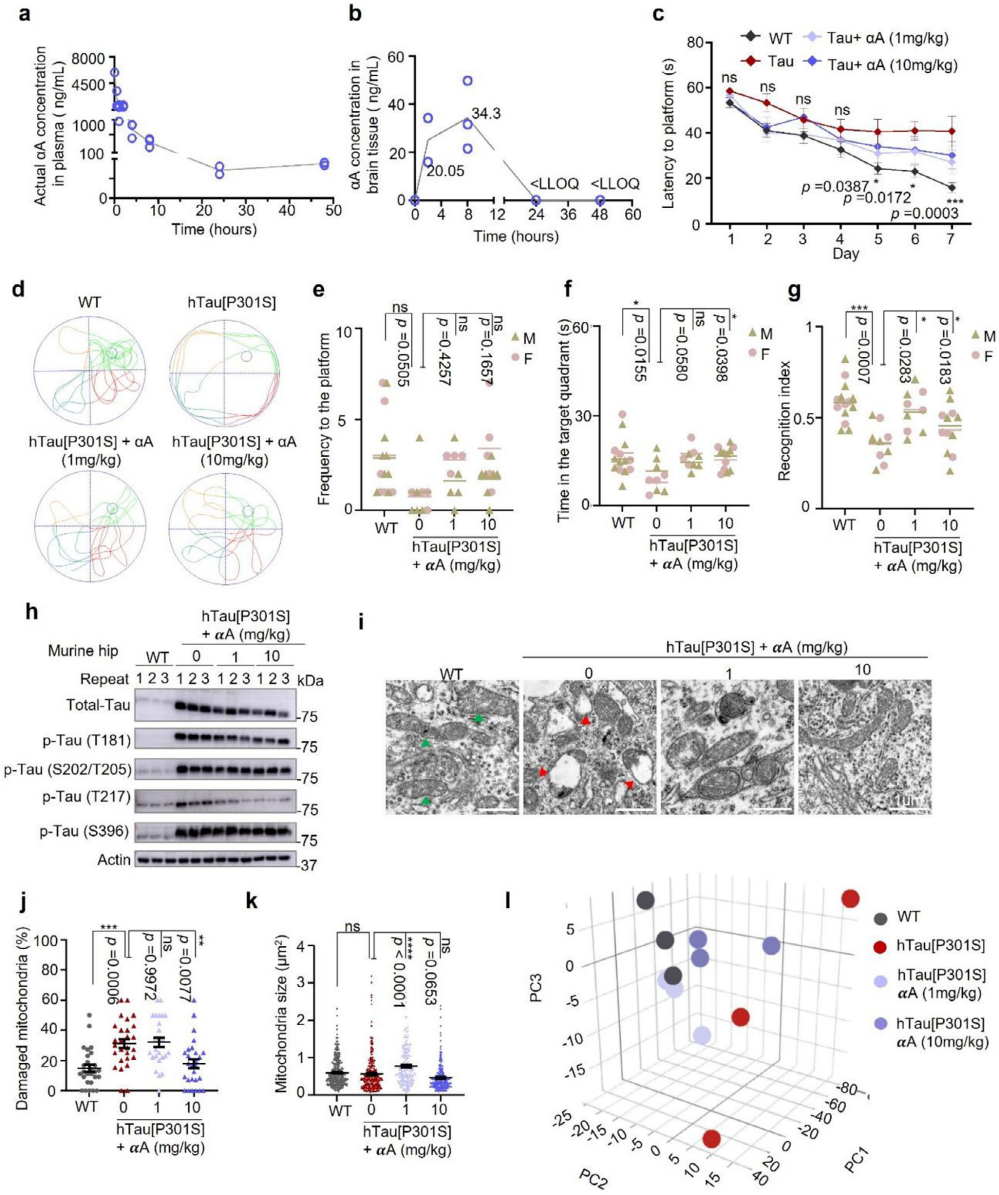
α‐Amyrin demonstrates favorable pharmacokinetics in WT mice and mitigates cognitive and pathological hallmarks in hTau[P301S] mice. (a,b) CD‐1 mice (*n* = 2–3) were dosed with a bolus intravenous injection of αA (10 mg/kg). Blood samples were collected 5 mins, 30 mins, and 2, 4, 8, 24, and 48 h after injection (a). Animals were sacrificed and brain tissue isolated 2, 8, 24 and 48 h after injection (b). Plasma and brain concentrations of αA were determined by LC‐MS/MS. *<LLOQ: sample concentration below the lower limit of quantitation. (c) Morris water maze test from 1st to 7th day. Latency to platform was measured in WT and hTau[P301S] mice in the absence (vehicle) or presence of αA (1 or 10 mg/kg). (d) Representative 60 s swimming tracks of mice performing the Morris water maze test on the 8th day. (e,f) Frequency to the platform (e) and time in the target quadrant (f) of WT and hTau[P301S] mice in the absence (vehicle) or presence of αA (1, 10 mg/kg) performing the Morris water maze test on the 8th day. (g) 24 h novel object recognition (NOR) test for WT and hTau[P301S] mice in the absence (vehicle) or presence of αA (1, 10 mg/kg). (h) Western blot of total Tau and phosphorylated Tau variants as indicated (Thr181, Ser202/Thr205, Thr217, and Ser396) in hippocampal tissue of WT and hTau[P301S] mice (*n* = 3 mice/group) in the absence (vehicle) or presence of 1 or 10 mg/kg αA. (i) Representative electron microscopy micrographs of mitochondria in hippocampal tissue from WT and hTau[P301S] mice treated with vehicle or αA (1, 10 mg/kg). The green arrows indicate healthy mitochondria, and red arrows indicate the damaged mitochondria. (j,k) Visual assessment of mitochondrial morphology calculated as percent damaged (j) or relative size (k) in hippocampal tissue of WT and hTau[P301S] mice treated with vehicle or αA (1 or 10 mg/kg). (l) 3D PCA for gene expression profiles in hippocampal tissue from WT and hTau[P301S] mice treated with vehicle or αA (1 or 10 mg/kg) (*n* = 3 mice/group). Unless specified elsewhere, data are mean ± S.E.M. For the murine behavioral experiments there were at least 9 mice/group. Statistical analyses used were as follows: Repeated measures ANOVA by Tukey's multiple‐comparisons test (c); two‐way ANOVA followed by Tukey's multiple‐comparisons test (e, f, g); one‐way ANOVA followed by Tukey's multiple‐comparisons test (j, k). All panels: *n.s*., not significant, *p < 0.05, **p < 0.01, ***p < 0.001, ****p < 0.0001.

Given the efficacy of αA/ βA in cells, the next step was to select an appropriate model with which to test pharmacokinetics in a living organism. It is important to determine αA's pharmacokinetic profile and whether it crosses the blood‐brain barrier (BBB) in order to ascertain whether αA could potentially be used to treat human disease in the central nervous system (CNS). As a first evaluation, 6.5‐week‐old healthy CD‐1 male mice were given 10 mg/kg αA via bolus intravenous injection, and plasma was collected at 5 and 30 min, then at 2, 4, 8, 24, and 48 h after dosing. Animals were euthanized, perfused, and brain tissue was collected 2, 8, 24 and 48 h after dosing. Samples were analyzed and αA was quantified using Liquid Chromatography‐Tandem Mass Spectrometry (LC‐MS/MS). The plasma concentration of αA peaked at 5930 ng/mL 5 min after injection and decreased to 87.4 ng/mL 48 h after injection. The volume of distribution (Vss) was 9.54 L/kg (Table S2), plasma elimination was biphasic, the terminal half‐life (*t*_1/2_) was 10.1 h, and plasma clearance rate was 11.1 mL/min/kg (Figure 4a). Crucially, αA was detected in brain samples at 20.1 ng/mL 2 h after injection, peaked at 34.3 ng/mL 8 h after injection, and was cleared from the brain slowly over 24 h (Figure 4b). These results demonstrate that αA exhibits competitive pharmacokinetics and crosses the BBB in mice. This, in turn, encouraged us to further investigate whether αA is able to improve cognitive functions in AD like mice.

### 2.5 α‐Amyrin Improves Memory and Reduces Tau Pathology in hTau[P301S] Mice

We next explored the effects of αA on behavioral and cognitive functions in hTau[P301S] [31] and WT control mice. Specifically, hTau[P301S] heterozygous mice were treated with vehicle or αA (1 or 10 mg/kg) via oral gavage for two consecutive months starting at 9.5 months. We observed lower and declining average body weight in hTau[P301S] (Veh.) mice (32.9, 31.1, and 29 g) as compared to WT mice (36.8, 38, and 38.9 g) at 9.5, 10.5, and 11.5 months of age, respectively; αA slightly reduced the loss of body weight in hTau[P301S] tauopathy mice (Figure S2a). Although vehicle‐treated hTau[P301S] mice showed deficits in memory and learning compared to WT mice, memory was significantly restored in hTau[P301S] mice treated with 1 or 10 mg/kg αA (Figure 4c–g). The Morris water maze test was used to test memory. Animals received training 3 times per day for 7 consecutive days (Figure 4c) and were subjected to a trial on the eighth day (Figure 4d). With no observable effects on swimming speed (Figure S2b) or frequency of platform crossings (Figure 4e), hTau[P301S] mice treated with vehicle spent 9.8 ± 1.8 s in the target quadrant, while αA‐treated (1 mg/kg, 10 mg/kg) hTau[P301S] mice spent 15.7 ± 1.1 or 16 ± 1.1 s in the target quadrant, respectively; WT mice spent 16.4 ± 1.7 s in the target quadrant (Figure 4f), indicating that αA significantly improved long‐term spatial memory in hTau[P301S] mice. In addition, hTau[P301S] mice showed 29.5% lower spatial working memory capacity in the Y‐maze test (Figure S2c), and 29.4% reduced visual recognition of novel objects in the 24 h novel object recognition (NOR) test (Figure 4g) relative to WT mice. Spatial memory (Figure S2c) scores increased 17.7% or 13.8%, and visual recognition scores increased 25.4% or 12.4% (Figure 4g) in hTau[P301S] mice treated with 1 or 10 mg/kg αA, respectively relative to untreated hTau[P301S] mice. The benefits are consistent in both male and female mice.

Τhe hippocampal region of the brain plays a critical role in learning and memory [32]. Therefore, we quantified expression of total Tau and tau levels at different p‐Tau sites (Thr181, Ser202/Thr205, Thr217, and Ser396) in hippocampal tissue from WT and hTau[P301S] mice treated with vehicle, 1 or 10 mg/kg αA using Western blot. Compared to WT (Veh), total Tau and p‐Tau phosphorylated at Thr181, Ser202/Thr205, Thr217, or Ser396 increased 94.2, 102.7, 81.4, 53.5, or 80.3%, respectively, in hippocampal tissue from hTau[P301S] mice (Figure 4h; Figure S2d). Similar to the in vitro experiments, in hTau[P301S] mice treated with 1 or 10 mg/kg αA, expression of total Tau in hippocampal tissue decreased 36.4% or 50.1%, respectively; p‐Tau (Thr181) decreased 36% and 36.6% and p‐Tau (Thr217) decreased 60.5% and 83%, respectively, but p‐Tau (Ser202/Thr205) and p‐Tau (Ser396) did not decrease in response to αA (Figure 4h; Figure S2d). These data suggest that αA improves cognitive function and reduces p‐Tau‐related pathology (Thr181 and Thr217) in a mouse model of tauopathy.

### 2.6 α‐Amyrin Promotes Mitochondrial Health and Normalizes Gene Expression in hTau[P301S] Mice

Given the positive correlation between Tau aggregation and mitochondrial dysfunction, we examined whether αA alleviates p‐Tau formation due to mitigation of mitochondrial damage. Homeostasis of the mitochondrial pool requires a balance between biogenesis and the selective degradation of faulty mitochondria via mitophagy [33]. In the context of AD, neurons have been reported to accumulate damaged mitochondria [22] and to exhibit metabolic changes associated with mitochondrial dysfunction [34]. We analyzed mitochondrial shape and size in the hippocampal region of WT and hTau[P301S] mice treated with vehicle or αA (1 or 10 mg/kg) using electron microscopy (EM); EM data showed two times more damaged mitochondria in the hippocampal tissue of hTau[P301S] mice than WT (Figure 4i–k). When hTau[P301S] mice were treated with 1 mg/kg αA, mitochondrial size and shape were restored (Figure 4j,k; Figure S2e,f), and upon treatment with 10 mg/kg αA, 42.8% of the damaged mitochondria were eliminated (Figure 4i,j). To further dissect the mechanisms by which αA reduces p‐Tau and damaged mitochondria, we analyzed differential gene expression in hippocampal tissue from WT and hTau[P301S] mice under different treatment conditions (αA 1 or 10 mg/kg) via RNA‐seq. Principal component analysis (PCA) showed clear separation between WT and hTau[P301S] gene expression profiles; the distance between the two groups was reduced after treatment with αA (Figure 4l). Specifically, 393 genes were differentially expressed (DEGs) (*p* < 0.05) in hTau[P301S] mice relative to WT mice (Figure S2g). Up‐ and down‐regulated DEGs (Figure S2g, left panel) were grouped into 8 clusters based on GO functional enrichment (Figure S2g, right panel) as follows: cluster 1 (*n* = 82 genes), FOXO3 signaling; cluster 3 (*n* = 101), neuroinflammation; cluster 4 (*n* = 106), immune system activation; cluster 5 (*n* = 65), synaptic integrity; cluster 6 (*n* = 63), stress response; cluster 7 (*n* = 66), extracellular matrix (ECM) remodeling; cluster 8 (*n* = 98), angiogenesis. Cluster 2 (*n* = 57) was not associated with any GO functional enrichment. This analysis sheds light on the effects of αA on mitochondrial function, revealing possible mechanisms by which αA could maintain the neuronal microenvironment and promote mitochondrial health in the hippocampus.

### 2.7 α‐Amyrin Fuels Mitochondria and Stimulates Cellular Mitophagy

**FIGURE 5:**
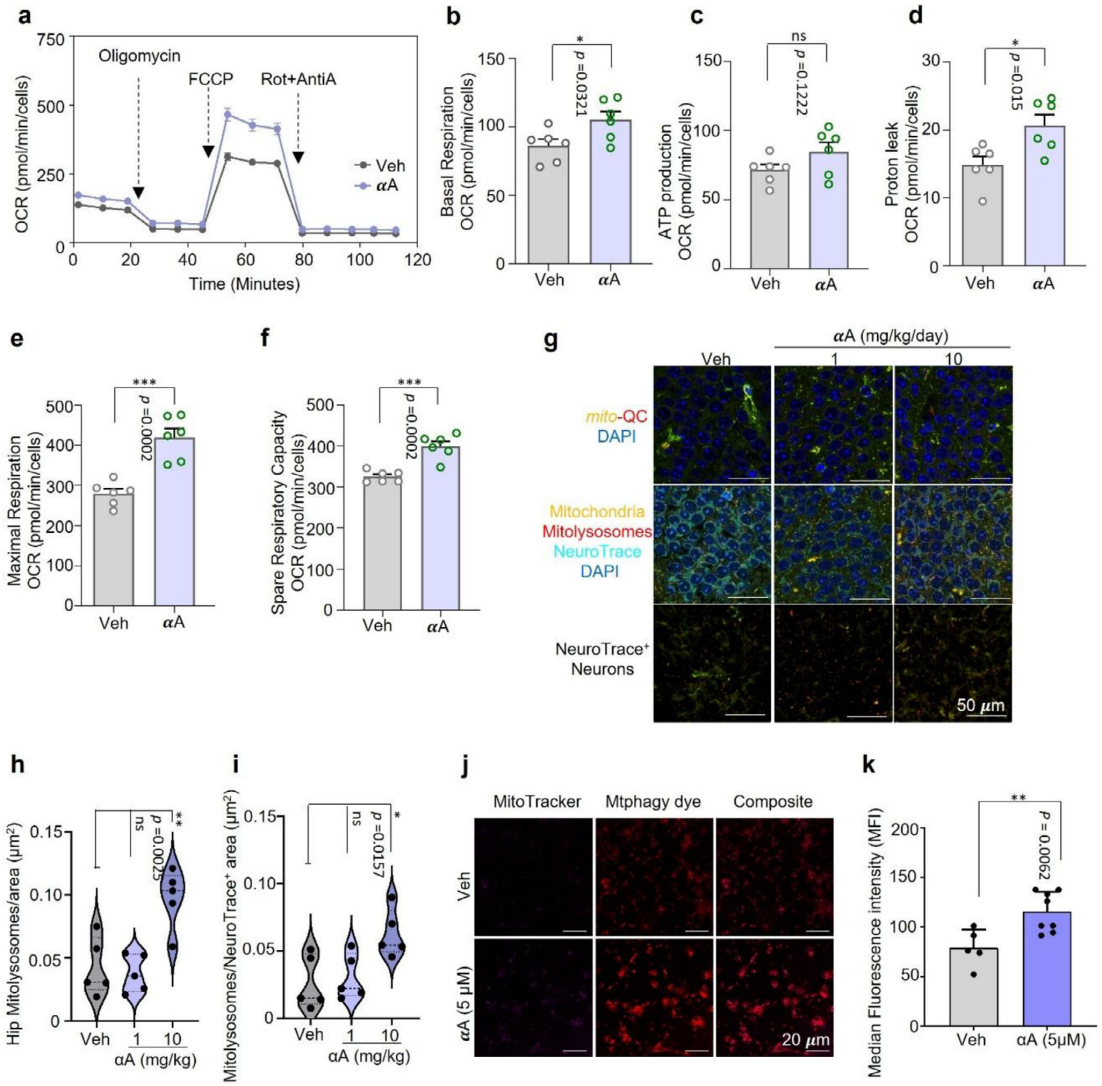
α‐Amyrin improves mitochondrial function and stimulates mitophagy in experimental animal models. (a) Real time basal oxygen consumption rate (OCR) was measured in ARPE‐19 cells treated with αA (1 µm) for 48 h with sequential injection of Oligomycin (1 µm, complex 5(CV) inhibitor), FCCP (1 µm, uncoupler) and Rotenone + Antimycin A (1 µm, CI+CIII inhibitors). Analysis was performed using a Seahorse XFe24 analyzer. Data points were normalized to number of cells. (b–f) Effect of αA (1 µm) on basal respiration (b), defined as the difference between basal OCR and non‐mitochondrial oxygen consumption; ATP production (c), defined as basal respiration minus sum of non‐mitochondrial oxygen consumption; proton leak (d) defined as basal respiration independent of ATP production; maximal respiration (e), defined as the difference between maximal (uncoupled) and basal respiration; spare respiratory capacity (f), defined as the difference between maximal (uncoupled) and basal respiration (a), respectively. (g) Representative images of brain cryosections of C57BL6/J mito‐QC reporter mice injected daily for two days with 1 or 10 mg/kg αA or vehicle. Cryosections were immunostained for cell type‐specific markers, neurons (NeuroTrace), and nuclei were counterstained with DAPI. Scale bar= 50 µm. (h,i) Quantitation of effects of αA (1, 10 mg/kg) based mitophagy induction in hippocampus (h), and neurons as described for (g). (j,k) Representative images of MitoTracker and Mtphagy dye‐stained human iPSC differentiated neural stem cells (hNSCs) (j) with quantification in (k). hNSCs were treated with αA (0, 5 µm). Unless specified elsewhere, data are mean ± S.E.M. Statistical analyses used were as follows: unpaired t‐test (b–f, k); one‐way ANOVA followed by Dunnett's multiple comparisons test (h,i); All panels: ns, not significant; * *p* < 0.05, ** *p* < 0.01, *** *p* < 0.001, **** *p* < 0.0001.

In addition to the morphological and size evaluation of mitochondria, we further investigated how αA affects mitochondrial functions such as the oxygen consumption rate (OCR) using standard Seahorse analysis in human retinal pigment epithelial ARPE‐19 cells, which originate from the neuroectoderm and are the most metabolically active cells in the body, relying heavily on mitochondrial function. Without or with exposure to respiratory stress (Figure 5a) (i.e., after sequential addition of oligomycin, carbonyl cyanide‐p‐trifluoromethoxyphenylhydrazone (FCCP), and rotenone and antimycin A; oligomycin limits respiration by blocking ATP synthase activity, FCCP collapses the mitochondrial membrane gradient for measurement of maximum OCR, and rotenone and antimycin A were added to measure minimum OCR) in the absence (vehicle) or presence of αA (1 µM), αA increased basal OCR by 22.2% (Figure 5a,b), ATP production by 18.6% (Figure 5c), proton leak by 39.3% (Figure 5d), maximal respiration rate by 50%, and reserve respiratory capacity by 22.8% (Figure 5a,e,f). These data suggest that αA increases basal mitochondrial function as well as resilience in response to stressors.

We next queried how αA improves mitochondrial homeostasis and function (Figure S3a–d) by exploring the status of mitophagy and autophagy, key processes for eliminating damaged mitochondria. ARPE‐19 cells stably expressing MAP macroautophagy reporter (GFP‐mCherry‐MAP1LC3B) or *mito*‐QC mitophagy reporter (GFP‐mCherry‐FIS1^101–152^) were used to quantify autophagy and mitophagy in the presence of vehicle or αA. Autophagy increased 4.2%, 22%, or 20% after 24 h treatment with 0.1, 1, or 10 µm αA, respectively (Figure S3a,b) and mitophagy increased approximately 50% after 48 h treatment with 1 or 10 µm αA (Figure S3c,d), with no change in mitochondrial membrane potential (ΨΔm) (Figure S3e) or cellular ROS (Figure S3f). These results suggest that αA induces mitophagy without depolarizing the mitochondrial membrane.

Since αA appears to have potential as an anti‐AD drug, we moved to assess its effects in a live model. The hippocampal brain region of C57BL6/J *mito*‐QC reporter mice was evaluated after αA (1 or 10 mg/kg) or vehicle treatment given via intraperitoneal injection for two consecutive days. αA given at a dose of 10 mg/kg was able to increase mitophagy 2.3‐fold overall in the hippocampus (Figure 5g,h), but did not induce any effect at 1 mg/kg. Interestingly, our results suggest αA's primary effect lies in neurons, where it increased mitophagy 2.4‐fold (Figure 5g,i) whereas it did not induce significant changes in either astrocytes or microglia at the tested doses (Figure S3g–i). To examine whether these effects carried over into human neural cells, human iPSC‐differentiated neural stem cells (hNSCs) with high mitochondrial activity and a well‐preserved autophagy–mitophagy network were used to further validate the neuronal mitophagy induction potential of αA; a 39.3% increase mitophagy was detected in αA (5 µm) treated hNSCs (Figure 5j,k) relative to vehicle.

### 2.8 α‐Amyrin Binds to and Inhibits Dual Leucine Zipper Kinase (DLK) and Normalizes Tau Pathology via DLK‐SARM1‐ULK1‐Mediated Mitophagy

**FIGURE 6:**
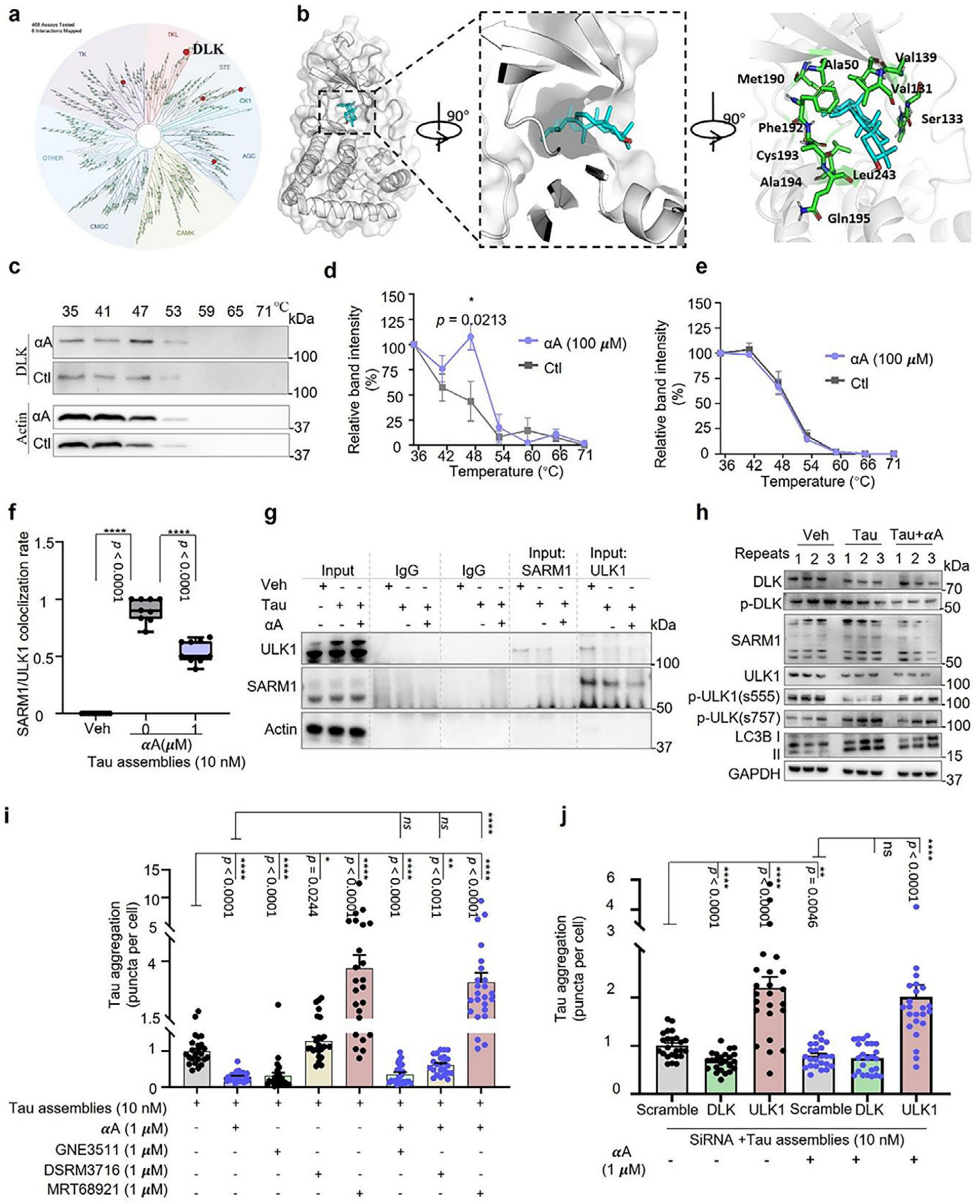
Integrated analyses identify and validate α‐Amyrin as a DLK‐binding compound. (a) Analysis of binding between αA (1 µm) and 468 commercially available kinases; red circles identify potential binding interactions, and circle size corresponds to binding affinity. (b) 3D representation of induced fit docking of αA and DLK based on prior structure analysis of DLK; DLK shown in gray, binding domains in green, and αA in blue. (c–e) Representative Western blot results of thermal shift assay (c), and quantification of αA‐induced shift in DLK thermal stability (d) and actin thermal stability (e). (f) Co‐localization of SARM1 and ULK1 in HEK293 P301S Tau‐Venus cells in the absence or presence of Tau seeds (10 nm) with or without αA (1 µm, 72 h). (g) Reciprocal co‐IP of SARM1 and ULK1 in HEK293 P301S Tau‐Venus cells with or without αA (1 µM, 72 h) treatment after Tau seeding. Input and IgG controls are shown. (h) Western blot of the indicated proteins in HEK293 P301S Tau‐Venus cells treated with vehicle or αA (1 µm, 72 h). (i) Tau puncta formation in HEK293 P301S Tau‐Venus cells in the presence or absence of DLK inhibitor GNE3511, SARM1 inhibitor DSRM3716, or ULK1 inhibitor MRT68921 treatment with αA (1 µm, 72 h). (j) Tau puncta formation in HEK293 P301S Tau‐Venus cells after SiRNA knock down of DLK or ULK1 genes. Cells were treated with vehicle or αA (1 µm, 72 h) as indicated. Unless specified elsewhere, data are mean ± S.E.M from 3 biological repeats. Statistical analyses used were as follows: Repeated measures ANOVA by Tukey's multiple‐comparisons test (d, e); one‐way ANOVA followed by Dunnett's multiple comparisons test (f); one‐way ANOVA followed by Tukey's multiple‐comparisons test (i, j). All panels: *n.s*., not significant, *p < 0.05, **p < 0.01, ***p < 0.001, ****p < 0.0001.

The above data indicated that αA improved cognitive function in mice, and that its effect is linked to ameliorated p‐Tau accumulation with improved mitochondrial function such as mitophagy. We therefore wished to identify the mode of action of αA to determine how and to which molecule(s) it was binding to create these improvements. Several kinases are involved in both Tau phosphorylation and mitophagy, so we used the KINOMEscan screening platform to evaluate interactions between αA (1 µm) and a list of 468 human kinases including disease‐relevant mutant variants. This platform determines whether the substrate (αA) binds to the kinase active site and directly or indirectly inhibits binding of the kinase to an immobilized ligand. A selectivity score of 35 (S35) was used as a potency threshold. The results show that αA binds 6 of 403 non‐mutant kinases, including DLK, dual‐specificity mitogen‐activated protein kinase kinase 4 (MEK4), Janus kinase 2 (JAK2), ribosomal protein S6 kinase (RSK4), mitogen‐activated protein kinase kinase kinase 15 (MAP3K15), and phosphatidylinositol 4‐kinase beta (PIK4CB). The most potent effect was on DLK (9.6% control) (Figure 6a) and so Schrödinger software was used to perform induced fit docking with αA as ligand for DLK which generated 10 putative high affinity docking conformations ranging from −7.973 to −9.878 kcal/mol (Table S3, Supporting Information). Notably, docking studies suggest that αA could bind preferentially with high affinity to hydrophobic regions of DLK and significantly alter the conformation of DLK's ATP‐binding site (Figure 6b). We hypothesized that the hydrophobic nature of the αA‐DLK interaction could play a critical role in the favorable docking of αA, a concept that is supported by the absence of conventional hydrogen bonding, typically a key feature in kinase inhibitor interactions. To validate whether αA directly binds to DLK, we assessed temperature‐dependence of binding over the range 35 to 71°C, showing that αA alters the thermal stability of DLK, especially at 47°C, but has no effect on thermal stability of actin (Figure 6c,d).

Interestingly, DLK (MAP3K12) is a neuron‐enriched mitogen‐activated protein kinase kinase kinase, that appears to be an essential regulator of neurodegeneration [35]. In response to cellular stress, activated DLK induces SARM1‐dependent neurodegeneration [36]; during this process, ULK1 is recruited and physically binds to SARM1 via its SAM (sterile α motif) domains [37], and is unable to initiate mitophagy [38]. Therefore, we asked whether SARM1 and ULK1 recruit and restrict activity mutually in the Tau assembly‐seeding environment, and whether αA inhibits Tau aggregate formation via the DLK‐SARM1‐ULK1 axis. A series of experiments were carried out in HEK293 Tau‐Venus cells in the absence or presence of Tau assemblies and αA; co‐localized SARM1 and ULK1 were found in immunofluorescent stained images at points where Tau puncta were located but were 36.5% lower in the αA treated group (Figure 6f; Figure S4a). Co‐immunoprecipitation (co‐IP) was performed to further evaluate the physical interaction of SARM1 and ULK1. Interestingly, we found SARM1 in the ULK1 pulled‐down precipitate but not the other way around, while the expression of both proteins was reduced in the αA treated group (Figure 6g). Next, since phosphorylation switches on activation of ULK1, and the activated ULK1 participates in mitophagy [38] we asked whether ULK gains activation when it is released from SARM1. The expression levels of DLK, p‐DLK (Thr43), SARM1, ULK1, p‐ULK1 (Ser555), p‐ULK1 (Ser757), and LC3 were detected using Western blot. Compared to WT, expression of SARM1, ULK1, p‐ULK1 (Ser757) and the ratio of LC3 II/I in the Tau assemblies‐seeded cells were increased by 40.9%, 19.8%, 67.6%, and 28.2% respectively, while p‐DLK (Thr43) and p‐ULK1 (Ser555) were decreased by 29.6% and 40.0%. However, this was modified in the presence of αA where increases in expression of DLK (12.4%), ULK1 (15.5%), p‐ULK1 (Ser555) (27.8%), and the ratio of LC3 II/I (38.2%) were all noted. The expression of p‐DLK (Thr43), SARM1, and p‐ULK1 (Ser757) were reduced by 54.1%, 45.5%, and 27.6%, respectively in the αA treated group when compared to the Tau assemblies seeded group (Figure 6h; Figure S4b). These data indicate that αA enhances ULK1 activation by inhibiting DLK activation, releasing ULK1 from co‐localization with SARM1.

To further investigate these mechanisms, mitophagy induction and Tau aggregate inhibition of αA were validated in the presence or absence of DLK (GNE3511), SARM1(DSRM3716), or ULK1 (MRT68921) inhibitors. The results in ARPE‐19 mt‐QC cells suggest that DLK and ULK1 are required for basal and αA‐induced mitophagy, but not SARM1 (Figure S4c). In the Tau seeded HEK293 Tau‐Venus cells, DLK inhibition (1 µm) and αA (1 µm) treatment dramatically inhibited Tau aggregate formation independently but had no additive effect. The situation was the opposite in SARM1 and ULK1 inhibitor (1 µm) treated cells, especially where the ULK1 inhibitor treatment (1 µm) heavily boosted Tau aggregate accumulation; the inhibitory effect on Tau aggregation was abolished upon αA treatment. To further validate these findings, HEK293 Tau‐Venus cells were treated with DLK and ULK1 SiRNA after confirming efficient DLK or ULK1 knockdown (Figure S4d,e, Supporting Information), followed by Tau assembly seeding and αA treatment. In line with the findings in the DLK and ULK1 inhibitor treated groups, there were no additional inhibitory benefits for αA treated DLK^KD^ cells, while the inhibitory effect of αA on Tau aggregation was abolished in ULK1^KD^ cells (Figure 6j). Collectively, we propose that binding of αA to DLK reduces the SARM1‐ULK1 complex, which activates ULK1‐dependent autophagy/mitophagy leading to Tau aggregation inhibition.

### 2.9 Elevated p‐Tau217 in Postmortem Human AD Hippocampus Is Inhibited by α‐Amyrin in 3D human AD‐Like Microfluidic Platform

**FIGURE 7:**
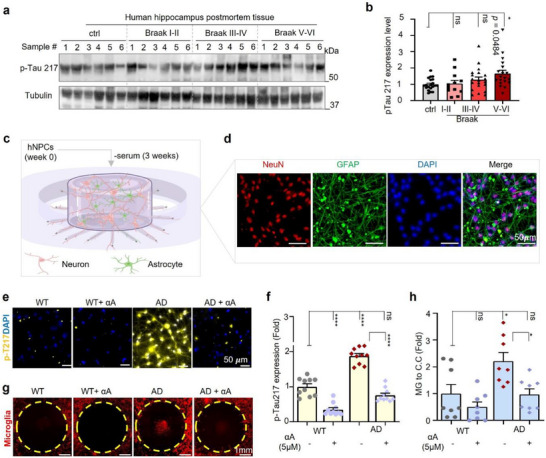
Data validation in human AD postmortem tissue and a physiological 3D AD‐like microfluidic platform. (a,b) Western blot (a) and corresponding bar graphs (b) to quantify expression of p‐Tau Thr217 in human hippocampal postmortem tissue. Samples are from normal controls or patients with Braak stage disease as indicated; *n* = 20 samples/group for control, Braak III‐IV (moderate AD), and Braak V‐VI (severe AD), and *n* =12 samples/group for Braak I‐II (mild AD). (c,d) Schematic diagram illustrating a maturated physiological 3D human brain on chip from hNPCs (c); immunofluorescent images confirmed the presence of neurons (NeuN, red), reactive astrocyte (GFAP, green) population, and nuclear (DAPI, blue). Scale bar = 50 µm. (e,f) Human 3D WT and AD microfluidic platform with vehicle or 5 µm αA treatment and immunostaining for p‐Tau Thr217 (e). Normalized fold change is shown in the bar graph of panel (f). Scale bar= 50 µm. (g,h) Effects of αA (5 µm) on infiltration of microglia in human WT or AD 3D microfluidic platform. Representative images show recruitment (48 h) of microglia (MG, red) to the central chamber (C.C.) in the absence or presence of αA (5 µm) (g). Bar graph of panels is shown in (h). Scale bar = 1 mm. Unless specified elsewhere, data are mean ± S.E.M. Statistical analyses used were as follows: one‐way ANOVA followed by Dunnett's multiple comparisons test (b). Two‐way ANOVA followed by Tukey's multiple‐comparisons test (f, h). All panels: *n.s*., not significant, *p < 0.05, ****p < 0.0001.

The above results from different models suggested αA alleviates Tau aggregation by inhibiting phosphorylation of Tau, especially p‐Tau217. Plasma p‐Tau 217 (phosphorylated at Thr217) has been proposed as a novel biomarker for AD [39, 40, 41], but data on p‐Tau 217 in human brain tissue across Braak stages is lacking. Braak staging (I‐VI) classifies AD progression based on the severity and spread of tau‐related neurofibrillary tangles. The later Braak stages reflect more advanced tau pathology and are strongly associated with greater cognitive impairment [42]. To address this gap, the expression level of p‐Tau 217 was quantified through Western blot analysis of human postmortem hippocampal tissue from normal controls (*n* = 20) or patients with mild cognitive impairment (Braak stages I‐II; *n* = 12), moderate cognitive impairment (Braak stages III‐IV; *n* = 20), or severe cognitive impairment (Braak stages V‐VI; *n* = 19). Western blot data showed 23.0% and 59.1% more pTau 217 in Braak stages III‐IV and V‐VI, respectively, compared to Braak stage I‐II (Figure 7a,b). To validate whether αA has the potential to reduce p‐Tau 217 in the human brain, we used a proxy 3D culture system to study Tau pathology [43]. This system features simultaneous tri‐culture of human neurons and astrocytes derived from human neuronal progenitor cells (hNPCs) (Figure 7c,d) and microglia [44] in a chemotactic chip. The health hNPCs were used for the wild type (WT) model development, while commercially available APPSL‐GFP Alzheimer's lentiviruses transduced hNPCs were used to develop AD model [45]. At microfluidic platform development period week 6, through the co‐immunostaining of neuronal marker (Tuj1), astrocyte marker (GFAP), and p‐Tau 217 antibodies, we found that the number of neurons was reduced by 11.4% while astrocyte reactivity was increased by 240% in the central chamber (C.C) in the vehicle treated AD platform when compared to the WT model (Figure S4f,g,h, Supporting Information). Three weeks αA (5 µm) treatment able to prevent neuronal loss and keep astrocytes in an inactivated state in AD platform (Figure S4f,g,h, Supporting Information). At microfluidic platform 9‐week stage, we further evaluate the neurotherapeutic potential of αA, and the immunostaining data exhibited that hyperphosphorylated p‐Tau 217 was 1.9‐fold higher in the vehicle treated AD model in comparison to the WT model (Figure 7e.f). Qualitatively, we observed 111.7% lower p‐Tau 217 in AD cultures treated with αA (5 µm, 6 weeks) when compared to vehicle‐treated controls (Figure 7e,f). Furthermore, we seeded human adult microglia to the annular chamber (A.C) in each microfluidic platform and evaluated the condition of microglia migration after 2 days. Upon on the interaction of neurons and reactive astrocytes in the C.C, the recruitment of microglia from A.C to the C.C was 2.2‐fold raised in a region‐specific manner in the vehicle‐treated AD platform when compared to the WT platform, but microglial migration was fully abolished in the αA (5 µm) treated AD platform (Figure 7g,h). These findings demonstrate that αA reduces the level of p‐Tau 217 in a 3D‐cultured human system comprising neurons and astrocytes and relieves microglia migration.

## 3 Discussion

We have evaluated the relationship between dementia risk, plasma p‐Tau217 level, and AFVC in a 10‐year clinical longitudinal cohort study, and we have shown that higher AFVC is correlated with lower rates of dementia [14, 15]. To uncover molecular mechanisms underlying the brain‐protective effect of AFVC, we integrated food‐omics, AI‐based computational methods (to screen anti‐AD candidates), and a series of experimental models (including cells, mice, human postmortem tissue, and a human 3D‐like microfluidic platform for functional validation), identifying αA as our lead compound. Over the past few decades, natural compound‐based drug discovery for AD has been a topic of great interest; many studies have focused on primary enriched plant metabolites such as phenolic compounds like flavonoids, but with limited success due to drawbacks such as poor stability and restricted brain bioavailability [46, 47]. Compounds with more lipid‐like features, enabling better membrane permeability, and higher metabolic stability show more promise. Therefore, triterpenoids, a specific subclass of terpenoids and the second most common plant metabolites enriched in plant‐based diets [48, 49], attracted our attention. Here, we identified the easily obtained pentacyclic triterpenol αA, a molecule found in many fruits and vegetables, as a lead compound for further investigation; it demonstrated competitive translational potential against AD, including robust anti‐AD properties and favorable pharmacokinetics (*T*_max_ = 0.083 h, and half‐life = 10.1 h) in preclinical studies.

Compromised mitophagy results in accumulation of damaged mitochondria, leading to mitochondrial senescence and apoptotic cell death, as well as induction of both the NLRP3‐IL‐1β and mitochondrial DNA/RNA‐cGAS‐STING inflammatory pathways, all of which are linked to AD risk and progression [50]. Genetic and pharmacological restoration of mitophagy inhibits these risk factors leading to a lower burden of Aβ and p‐Tau pathologies and memory retention in pre‐clinical models of AD; several clinical trials have been completed or are on‐going in this area [22, 51, 52]. The concept of this study was to identify a fruit‐ and/or vegetable‐derived lipid‐like compound that was able to alleviate AD‐like pathologies via modification of cellular homeostatic functions like mitophagy, and for this compound to be tested as a potential drug candidate against AD. Here, to reduce false‐positives among the potential drug candidates, we employed a cross‐species platform to identify our lead compound, a technique we have previously used successfully [22, 23]. First, we found that αA eliminates accumulation of Tau aggregates and reduces p‐Tau, especially p‐Tau 217 in a Tau seeding system using HEK293 Tau‐Venus cells. We then used hTau[P301S] mice to evaluate the therapeutic potential of αA on Tau pathology and cognitive function as they develop progressive neurodegeneration and behavioral deficits that closely mimic AD‐related phenotypes in humans. In these mice, we found that αA improves cognitive functions such as short‐term memory with fewer damaged mitochondria and restoration of transcriptional status. Next, we used human retinal pigment epithelial ARPE‐19 and ARPE‐19 *mito*‐QC cells to evaluate how αA affects mitochondrial function and mitophagy, respectively. In addition, mito‐QC mice were used to detect αA's potential mode of action (neuronal mitophagy induction) in vivo because they express a dual‐fluorescent mitochondrial reporter (mCherry–GFP) that enables quantitative visualization of mitophagic flux, which cannot be directly assessed in conventional models. Very importantly, the anti‐AD potential of αA was preserved from animal models of AD to a physio‐pathological 3D human brain on chip (established previously [44]), and the data highlighted that αA comprehensively regulates the microenvironment including protection from neuronal damage and p‐Tau accumulation (such as p‐Tau217 formation) and abolished neuroinflammation. Mechanisms on how αA inhibit inflammation should be explored in depth in the future studies.

αA alleviates p‐Tau formation, and enhances stress resilience and mitophagy in cells, which may be affected by kinase regulation. Through kinase screening and computational ligand/kinase docking studies, we identified αA as a novel inhibitor of DLK. While more “conventional” small molecule inhibitors interact with the kinase's active site through hydrogen bonding between donors and acceptors via hydrophilic binding pockets, our data indicated that αA appears to selectively interact with hydrophobic residues in DLK, exploiting its steroidal backbone and hydrophobic cyclic structure. This allows αA to induce conformational changes in the kinase ATP‐binding site. DLK is a master regulator of axon degeneration in response to acute neuronal injury such as stroke and chronic neurodegeneration like AD [53]. Microtubule‐associated protein Tau is enriched in axons and is essential for microtubule structure and function. However, accumulation of p‐Tau causes neurite degeneration and viable synapse loss, leading to behavioral abnormalities [54]. Previous studies indicate that pharmaceutical or genetic inhibition of DLK attenuates neuronal death [55, 75], but the relationship between DLK and p‐Tau was not explored. SARM1 is a TIR motif‐containing a nicotinamide adenine dinucleotide (NAD^+^) nucleotidase involved in NAD^+^ metabolism, and activated SARM1 is a DLK target that participates in axon degeneration [36, 56]. A recent study demonstrated that ULK1 is recruited by SARM1 during neural damage [37]. It is well‐documented that ULK1 is essential for autophagy and mitophagy, and plays important roles in vesicle transport, axonal guidance and elongation [57]. Therefore, we speculated that sequestration of ULK1 into SARM1‐ULK1 complexes might lead to reduced autophagy/mitophagy due to less bioavailable ULK1. Our results show that DLK was activated in the HEK293 Tau Venus cells and additional ‘Tau assemblies’ increased occurrences of co‐localized SARM1 and ULK1 along with Tau aggregates. αA was able to uncouple SARM1 and ULK1, and limit cellular Tau aggregates via autophagic elimination of newly generated Tau aggregates and enhanced degradation of existing ones. Mechanistically, αA appears to eliminate AD pathologies via modulation of the DLK‐SARM1‐ULK1‐autophagy/mitophagy axis.

Pharmacokinetic data demonstrate that αA crosses the blood‐brain barrier (BBB) in mice which is an enormous advantage as more than 98% of small molecule CNS‐active candidate drugs do not [58]. Furthermore, αA has a high volume of distribution and relatively long half‐life (*t*_1/2_ = 10.1 h), indicating its capacity to distribute efficiently to peripheral tissues. A half‐life of around 12–48 h is generally ideal for once‐daily dosing of oral drugs [59] and the relatively slow clearance of αA may allow for less frequent dosing without loss of efficacy. BBB permeability studies conducted solely in one healthy mouse strain have limitations when extrapolating to human AD, and several factors need be considered: 1) the BBB is integral and highly selective in healthy mice, whereas it is often altered in AD mice due to amyloid/Tau pathology, neuroinflammation, and impaired active transport; the active uptake rate and passive diffusion situation may also change due to barrier leakage; 2) the species differences between mouse and human BBB further complicate immediate translation of our findings to a clinical trial. Human BBB has distinct expression patterns of transporters, receptors, and metabolic enzymes, which influence drug transport and clearance kinetics; and 3) age‐related changes to the BBB can influence drug penetration and efficacy in the aging and diseased brain. Therefore, future studies are required to investigate how αA crosses the blood‐brain barrier in healthy and AD mice of different ages, and to analyze its pharmacokinetics in humans (i.e., phase I clinical trials).

While our study has identified a very robust mitophagy inducer with high translational potential, we acknowledge some important limitations. First, as we reported before, the crude extract of passion fruit exhibited strong anti‐AD potential in AD‐like animal models, while αA is a lead compound in passion fruit [21], future studies are required to confirm whether αA is a primary contributor to the anti‐AD effects of passion fruit, and other fruits and vegetables. Second, it is a challenge to calculate how much αA was consumed per participant in the Shanghai Ageing Study cohort; more or less of the compound may have been consumed in the population. However, it is not possible to strictly control the intake of food in a community setting, so it is our belief that while this was a “best guess” scenario, it has provided us with good quality data from which to build our study. Second, since mitophagy is a sub‐pathway of autophagy, it is very likely that αA‐induced autophagy (excluding mitophagy) also contributes to its anti‐AD function; this should be studied further in future studies. Next, while αA strongly binds to and inhibits the activity of DLK, we cannot exclude the possibility that αA also binds to and/or inhibits other proteins contributing to its anti‐AD potential. More detailed molecular mechanisms on how αA induces mitophagy in vivo should be explored in the future, including to use conditional DLK‐knockdown (knockout) mice as well as the use pharmacological approaches in investigate the proposed and other potential pathways that the molecule may affect. This new data will propel any αA's translational applications. Further, as a plant metabolite, it is possible that αA may influence the gut‐brain axis, and this too will be important to evaluate in future studies. Finally, the clinical efficacy of αA in AD patients is still to be determined. Future studies will need to address how αA modifies both the cognitive and molecular components of AD and other neurodegenerative disorders.

This study reinforces that the rate of dementia onset is inversely correlated to the level of plant‐based food consumption; terpenoids like αA from colorful fruits and vegetables can pass through the BBB and improve cognitive functions offering a new, safe, and effective direction for neuroprotective drug development.

## 4 Materials and Methods

### 4.1 Reagents, Chemicals, and Antibodies

DMEM (1X) + GlutaMAX‐|Dulbecco's Modified Eagle Medium Mixture and Opti‐MEM (no FBS no antibiotics), DMEM:F12 (41966‐029, 21765‐029), Neurobasal Plus (A3582901), 1 mM GlutaMAX (35050061), Penicillin‐Streptomycin (P/S) (15140‐122), 1X B‐27 Plus (A3582801), L‐Glutamine (25030‐024) were purchased from Gibco by Life Technologies. Fetal bovine serum (FBS), penicillin‐streptomycin, Ampicillin, Thiazolyl Blue Tetrazolium Bromide, epidermal growth factor (EGF), α‐Amyrin (53017‐10MG‐F), and β‐Amyrin (09236‐10MG‐F) were from Sigma‐Aldrich. Dimethyl sulfoxide (DMSO), NaCl (39719, G7021, P1754, 1.06404.1000), Triton X‐100 (T8787), NGS (G9023), RIPA buffer (R0278) phosphatase inhibitors ((NaF), 201154), Na_3_VO_4_ (S6508), Na_4_P_2_O_7_ (221368) and protease inhibitor cocktail (P8783), total protein Ponceau S staining (78376) were purchased from Merck. TRIzol Reagent (Cat. #BCCD4264) was purchased from Sigma Life Science. NuPAGE 4–12% Bis‐Tris Midi Gels, CellROX Deep Red (C10422), TMRM (T668), Lipofectamine RNAiMAX, and Lipofectamine 2000 were purchased from Invitrogen, Thermo Fisher Scientific. NuPAGE MES SDS Running Buffer (20X) and NuPAGE Transfer Buffer (20X) were purchased from Invitrogen, Life Technologies. Scramble (AM4611), trypsin (11500636), TO‐PRO‐3 (T3605), DAPI (62248), TMRM (T668), Lipofectamine 3000 (L3000001), Hygromycin B (10687010), HEPES (15630‐080), Pierce BCA Protein Assay Kit (23227), Pierce ECL Western Blotting substrate (32106), StemPro NSC medium, anti‐rabbit‐Alexa 647 (A32733), NeuroTrace 640/660 (N21483) antibodies were purchased from Thermo Fisher. Film was purchased from AGFA. ULK1 (Human) – 3 unique 27mer siRNA duplexes (SR322391), MAP3K12 (Human) – 3 unique 27mer siRNA duplexes (SR305269) were purchased from OriGene. Vectashield Antifade Mounting Medium (H‐1000‐10) was purchased from Vector Laboratories. APPSL‐GFP Alzheimer's lentiviruses and LentiBrite GFP lentiviruses were purchased from EMD Millipore. Matrigel was purchased from BD Biosciences. Heparin was purchased from StemCell Technologies. B27 neural supplement was purchased from Life Technologies. Fibroblast growth factor‐basic (bFGF) was purchased from Stemgent. Human adult microglia (SV40) were purchased from Applied Biological Materials Inc. Seahorse XF24 Cell Culture Microplate (100882‐004), Seahorse XF DMEM Assay Medium (103680), Pyruvate (103578), and Glucose (103577) were purchased from Agilent. Criterion TGX Precast Stain‐free Gels (5678124), Immun‐Blot polyvinylidene fluoride (PVDF) Membranes for Protein Blotting (Cat. #1620177), Tween‐20 (1706531), and iScript cDNA Synthesis kit (Cat. #1708891) were purchased from BIO‐RAD. Additionally, Non‐fat dry milk, antibodies including Tau (D1M9X) (46687), p‐Tau (Thr181) (12885), p‐Tau (Ser199) (29957), ULK1 (D9D7) (6439), p‐ULK1 (Ser555) (D1H4) (5869), p‐ULK1 (ser757) (D7O6U) (14202), GAPDH (14C10) (2118) as well as anti‐rabbit IgG HRP‐linked antibody (7074) and anti‐mouse IgG HRP‐linked antibody (7076) were purchased from Cell Signaling. Antibodies including p‐Tau (Thr 217) (44‐744) and p‐Tau (Ser202‐Thr205) (MN1020), anti‐rabbit (31460), and anti‐mouse (31430) HRP‐conjugated secondary antibodies, and Geltrex, E8 medium were purchased from Invitrogen. Antibodies LC‐3B (NB100‐2220), MAP3K12 (NBP2‐17218) were purchased from NOVUS. SARM1 (55488‐1‐AP), MAP2 antibody (ab5392) was purchased from proteintech and Abcam, respectively. AT8 (MN1020), anti‐chicken 488 (A‐11039), anti‐mouse 647 (A21235) antibodies were purchased from Thermo Fisher. GFAP antibody (Z0334) was purchased from DAKO, Iba1 (019‐19741) was purchased from Wako. Mitophagy Detection Kit was purchased from Dojindo.

### 4.2 Calculation of Annual Fruit and Vegetable Consumption and Correlation Analysis With Dementia and Plasma p‐Tau217 Level

The annual fruit and vegetable consumption (AFVC) was calculated from the food frequency questionnaire (FFQ) data of the Shanghai Aging Study cohort (*n* = 1704). Participants aged 60 or older who were permanent residents in the community were administered the questionnaire at baseline to document self‐reported daily dietary intake over the preceding 12 months [60]. In brief, the FFQ involves 111 items including sugar, meat products, salted foods, oil, and fruits and vegetables. In the present study, the selected fruits and vegetables were used for AFVC as following:

$$\mathrm{AFVC}\left(\mathrm{kg}/\mathrm{year}\right)=\mathrm{times}\left(\mathrm{per}\mathrm{month}\right)\times g\left(\mathrm{per}\mathrm{time}\right)\times 1000\times 12$$

The Mini‐Mental State Examination (MMSE) and domain‐specific tests were conducted at baseline and follow‐up. Participants who met the DSV‐IV criteria were diagnosed with dementia and others were considered dementia‐free as reported in our previous study [16]. Here, the statistical difference between AFVC in the non‐dementia and dementia groups was analyzed using unpaired t test; the results were presented as mean and standard error of the mean (SEM). The Kaplan–Meier curve with Log‐rank test was performed for the cumulative incidence rate of dementia at follow‐up time in non‐dementia and dementia groups. In addition, overnight fasting blood samples were collected and stored at ‐80°C, and the plasma p‐Tau217 level of all participants at baseline was reported in our previous study [16]. Here, Pearson's correlation coefficient r was used to evaluate the relationship between the between the AFVC and plasma p‐Tau217 level at baseline. Tests were set at a significance level of 0.05 with two tails.

### 4.3 Fruit‐Compound Network Analysis and Potential Anti‐AD Compounds Prediction

Based on AFVC, a comprehensive search of FooDB (www.foodb.ca) and literature identified 240 fruit‐sourced candidate compounds. The list of edible fruits included tomato, apple, bell pepper, Asian pear, grape, eggplant, European grape, American cranberry, passion fruit, olive, and sweet cherry. The fruit compound network was built using Cytoscape 3.9.1. After identification of the target compounds sourced from fruit, we retrieved the compound‐protein binding partners from the databases ChEMBL [61] and Binding DB [62]. The biological activity categories include: (i) inhibitory constant (Ki), dissociation constant (K_d_), half maximal inhibitory concentration (IC_50_), biological potency, and half maximal effective concentration (EC_50_); (ii) the protein target is a human protein; (iii) the protein target is represented by a unique UniProt accession number; and (iv) the compound can be represented using a canonical SMILES format. All unique experimentally validated compound‐protein interactions were mapped to corresponding digital identifiers using InChIKey (https://www.inchi‐trust.org); in total, 1,144 partners were identified.

To examine the edible fruits compounds’ effects on AD, a proximity network analysis was used, based on a previous study (source code: https://github.com/ChengF‐Lab/COVID‐19_Map) [26]. Briefly, a nearest distance *d*(*S*, *T*) will be calculated between targets of fruit‐sourced compounds (*T*, 1,144 proteins) and AD‐related proteins (*S*, 144 AD seeding proteins) [27] to quantify how near the targets per compound are to AD on the potential in‐house protein‐protein interactions (*n* = 351 445):

$$d\left(S,T\right)=\frac{1}{\left\|S\right\|+\left\|T\right\|}\left(\sum_{t\in T}\underset{s\in S}{\min d}\left(s,t\right)+\sum_{s\in S}\underset{t\in T}{\min d}\left(s,t\right)\right)$$

where *d*(*s*, *t*) indicates the shortest path length between AD protein *s* and target of compound *t*. The randomization for each compound was performed and repeated 1000 times. Z‐score and *p* value were calculated to normalize the nearest distance and reduce the study bias:

$$Z=\frac{d\left(S,T\right)-\mu }{\sigma }$$

### 4.4 Cell Culture

HEK293 cells stably expressing the 0N4R isoform of human P301S mutant Tau with a C‐terminal Venus tag (HEK 293 P301S Tau‐Venus) were a generous gift from Dr. William A. McEwan at University of Cambridge, UK. Cells were cultured and maintained in DMEM with 10% FBS and 1% P/S in a humidified incubator at 37°C under 5% CO_2_. The medium was replaced every 2 to 3 days, and cells were harvested for experiments when they reached 50–80% confluency.

ARPE‐19 WT cells and ARPE‐19 stably expressing *mito*‐QC or MAP reporter were kindly provided by the group of Dr. Ian Ganley [63]. Cells were maintained in DMEM/F12 with 15% FBS, 1% P/S, and 2 mm L‐glutamine in a humidified incubator at 37°C under 5% CO_2_. *mito*‐QC cells were grown in the presence of 800 µg/mL Hygromycin B to ensure homogeneous positive selection of reporter‐expressing cells.

AD human neural progenitor cells (AD hNPCs) were derived from ReN cell VM human neural progenitor cells (EMD Millipore, Billerica, MA) by transduction with commercially available APPSL‐GFP Alzheimer's lentiviruses to produce high levels of Aβ, as described previously [45]. For controls, hNPCs were transduced with LentiBrite GFP lentiviruses. Both AD and control hNPCs were plated on 1% Matrigel in DMEM/F12 solid media containing 2 µg/mL heparin, 2% B27 neural supplement, 20 ng/mL EGF, 20 ng/mL bFGF, and 1% P/S and incubated at 37 °C supplied with 5% CO_2_. Culture medium was changed every 3 days until cells were confluent.

### 4.5 Human iPSC‐Differentiated Neural Stem Cells (hNSCs) Culture

Detroit 551 fibroblasts (ATCC CCL 110) were reprogrammed into induced pluripotent stem cells (iPSCs) using retroviral vectors encoding POU5F1, SOX2, Klf4, and c‐Myc, as previously described [64, 65]. The iPSCs at ∼70% confluence were replated on Geltrex‐coated plates. After 24 h, cultures were switched to chemically defined medium (CDM; 1:1 IMDM/F12 with GlutaMAX; plus BSA Fraction V, Lipid Supplement 100×, 1‐thioglycerol, insulin, and transferrin) containing SB431542 (10 µm), N‐acetyl‐L‐cysteine (10 µm), and AMPK inhibitor Compound C (2 µm). Medium was refreshed daily until neuroepithelial morphology was evident (day 5) [64]. Cells were loosened with collagenase IV, rinsed in DPBS^−/−^, and transferred to StemPro NSC medium with 1× GlutaMAX, bFGF, and EGF. Neurospheres were generated by gentle scraping and cultured in 100 mm non‐treated dishes on an orbital shaker at 37°C. After 2–3 days, spheres were dissociated with TrypLE Express (10 min, 37°C), neutralized in DMEM with 10% FBS, and replated as monolayer NSCs on Geltrex in StemPro NSC medium. NSCs at passages 4–9 was used for all downstream analyses. All cells were regularly monitored for mycoplasma contamination using the MycoAlert Mycoplasma Detection Kit

### 4.6 Cell Viability Assay

Cell viability was measured using the 3‐(4,5‐dimethylthiazol‐2‐yl)‐2,5‐diphenyltetrazolium bromide (MTT) assay as described previously. Briefly, HEK 293 P301S Tau‐Venus cells were seeded in 96‐well plates (5000 cells/well) and incubated for 24 h at 37°C under 5% CO_2_. Cells were treated with α‐Amyrin (αA) or β‐Amyrin (βA) (0.125 to 30 µm) for 48 h, MTT reagent was added, and cells were incubated for another 3 h. Cell pellets were collected, the supernatant was removed, and formazan crystals were dissolved in DMSO (200 µL). The absorbance was measured at 550 nm.

### 4.7 Assay for Tau Aggregation and Degradation in HEK 293 P301S Tau‐ Venus Cells

Exogenous recombinant heparin‐assembled Tau P301S (Tau assemblies) were generated at the William A. McEwan laboratory at Cambridge University, and used as previously described [28]. In brief, the HEK293 P301S Tau‐Venus cells were cultured in DMEM containing 10% FBS and 1% P&S in a 37°C humidified incubator under 5% CO_2_. On the day of the experiment, cells were trypsinised and seeded in 50 µL Gibco Opti‐MEM (without FBS and antibiotics) at a density of 5000 cells/well in a 96‐well plate (precoated with Poly‐D‐Lysine/PDL) and incubated for 24 h. Then, 50 µL Opti‐MEM + 1% Lipofectamine 2000 (no FBS no antibiotics) containing 20 nm Tau assemblies was pre‐incubated at room temperature for 10 min, added to each well, and incubated for 1.5 h. (1) To monitor Tau aggregation, 50 µL DMEM (with 20% FBS and 1% P&S) plus vehicle (2‐propanol), αA or βA was added to each well. The 96‐well plates were incubated for 72 h. Cells were then fixed with 4% formaldehyde in PBS for 10 min at room temperature, stained with DAPI (1 µg/mL) in PBS for 10 min and visualized using a ZEISS Axio Zoom.V16 microscope. (2) To quantify degradation of Tau, 50 µL DMEM (with 20% FBS and 1% P&S) was added to 96‐well plates, followed by 48 h incubation. The medium was replaced with 150 µL DMEM with 10% FBS, 1% P&S, and vehicle (2‐propanol l), αA, or βA (1, 5, or 15 µµ) respectively, for 24 or 48 h. Cells were fixed with 4% formaldehyde solution in PBS for 10 min at room temperature, stained with DAPI (1 µg/mL) in PBS for 10 min, and visualized under a ZEISS Axio Zoom V16 microscope. One image was taken per well. Eight technical repeats were performed per biological replicate. ImageJ was used for data analysis and the final results represent pooled data from 3 biological replicates. Tau puncta per cell are equal to the number of puncta divided by the total number of cells in each image; data were normalized to vehicle.

### 4.8 Tau Seeding Assay in Primary Neurons

Primary neuron cultures were prepared from transgenic mice expressing human Tau with frontotemporal lobar degeneration (FTLD) associated mutation P301S [31]. Brains were removed from post‐natal day zero or day one mice and pooled hippocampal and cortex cultures were prepared as previously described [66]. On day in vitro (DIV) six, αA or 2‐propanol (Vehicle) were diluted in neuron maintenance media composed of Neurobasal Plus, 1 mm GlutaMAX, 10% horse serum, 1% penicillin‐streptomycin and 1 X B‐27 Plus, and applied to cultures overnight. Compounds remained on the cells for the duration of the assay. On DIV seven, cultures were seeded with Tau assemblies, as previously described [66]. Briefly, 100 nm recombinant Tau assemblies were diluted in neuron maintenance media and applied to cultures. Cultures were incubated at 37°C and 5% CO_2_ until DIV14. On DIV fourteen, cultures were fixed with methanol and stained with MAP2 and phospho‐tau antibodies for immunofluorescence imaging [66]. Analysis was completed using NIS Elements version 5.41.02 software (Nikon). AT8 positive phospho‐tau puncta and cell bodies were quantified and divided by cell count determined via DAPI staining. All conditions were normalized to the seeded solvent control. Six images were taken per well, with three technical repeats across three biological replicates.

### 4.9 Genetic Modifications in Cells

To knockdown the target gene in cells, HEK 293 P301S Tau‐Venus cells were seeded in 6‐well plates (1.5 × 10^5^ cells/well) or 96‐well plates (precoated with Poly‐D‐Lysine/PDL, 5000 cells/well) and incubated for 24 h at 37°C under 5% CO_2_. For each transfection, cells were treated with siRNA at designed concentration (20, 40, or 80 nm) with 0.4% Lipofectamine RNAiMAX in total 1 mL Opti‐MEM for 6 h. The transfection was terminated by add 1 mL DMEM containing 20% FBS and 1% P&S. The media were replaced using DMEM containing 10% FBS and 1% P&S after 24 h. To evaluate knockdown efficiency, the cells were collected after 48 h, lysed, and the protein expression levels were detected via Western blot. To investigate whether knocking down the target gene at designed concentrations effects Tau aggregate formation, a Tau seeding assay was performed after a 48 h knockdown period

### 4.10 Co‐Immunoprecipitation (co‐IP)

The HEK 293 P301S Tau‐Venus cells were seeded in 10 cm plates (5 × 10^6^ cells/well) and incubated for 24 h at 37°C under 5% CO_2_. Cells were treated in Opti‐MEM with 20 nm Tau assemblies and 1% Lipofectamine 2000 for 1.5 h, and then αA (1 µm) treatment was introduced. After 72 h, cells were washed with ice‐cold PBS, and lysed n 500 µL trypsin, collected to pre‐labeled Eppendorf tubes, and resuspended in 100 µL hypotonic lysis buffer. The cell lysate was incubated on ice for 5 min followed by 3 snap freeze‐thaw cycles in liquid nitrogen and a 37°C water bath. The cell lysate was incubated with 140 mm NaCl and 0.5% NP‐40 on ice for 10 min. The samples were further diluted with 150 µL hypotonic buffer with 0.5% NP40 and incubated on ice for 20 min. The whole cell extracts were collected after centrifugation (12 000 x g, 10 min, 4°C). Protein concentration of each sample was detected by BCA assay and adjusted to 200 µg. For immunoprecipitation, 0.2 mg protein samples were incubated with 2 µg primary antibody or IgG in total 200 µL IP buffer at 4°C. After 24 h, the protein‐antibody mixtures were added to pre‐washed protein magnetic beads (dynabeads A for rabbit antibody; dynabeads G for mouse antibody, 25 µL) in low retention tubes. The samples were rotated 24 h at 4°C. The sample tubes were placed on a magnet and washed 3 times with ice‐cold IP buffer. The dynabeads‐antibody‐antigen pellets were resuspended in 1x NuPAGE LDS sample buffer with 4% DTT. The final precipitates were gently mixed and heated for 10 min at 95°C and detected by immunoblotting.

### 4.11 Evaluation of Mitochondria Respiration in ARPE‐19 Cells

Oxygen consumption rate (OCR) was measured using standard Seahorse technology. Briefly, 50 000 ARPE‐19 cells/well were seeded in a Seahorse XF24 Cell Culture Microplate in complete medium and incubated overnight at 37°C under 5% CO_2_. Cells were washed with PBS and culture medium was replaced with DMEM with 10% FBS, 1X Pen/Strep and vehicle (2‐propanol) or αA (1 µµ) and incubated for 48 h. Medium was replaced with Seahorse XF DMEM Assay Medium with 2 mm Pyruvate and 25 mm glucose. Oligomycin, FCCP and Rotenone+Antimycin A (1 µm) were injected sequentially and OCR was measured using the Seahorse XFe24 Analyzer (Agilent).

### 4.12 Quantification of Mitophagy, Autophagy, Mitochondrial Membrane Potential (ΔΨm) and Oxidative Stress by Flow Cytometry

ARPE‐19 mt‐QC cells were employed to evaluate whether αA could induce mitophagy and change ΔΨm in vitro. Briefly, ARPE‐19 mt‐QC cells were seeded in 24‐well plates at 5 × 10^4^ cells/well in DMEM/F12 with 10% FBS and 1% P&S and incubated for 24 h at 37°C under 5% CO_2_. Vehicle (2‐propanol) or αA (1, 5, 15 µµ) was added, cells were incubated for 24 or 48 h, then harvested, washed with PBS, trypsinized for 2–3 min and collected by centrifugation at 1200 × g for 5 min. Cells were resuspended and stained with DAPI (1 µg/µL). Samples were analyzed using a CytoFlex S (V4‐B2‐Y4‐R3; Beckman Coulter) flow cytometer. At least 10,000 events were collected. The viable population was selected by nuclear exclusion with 1 µg/µL of DAPI or TO‐PRO‐3. For *mito*‐QC analysis, GFP mean fluorescent intensity and mCherry/GFP ratios were calculated to estimate relative number of mitophagic events. A similar approach was used to study autophagy using MAP cells. Representative images were obtained using an AF6000 LX epifluorescence microscope (Leica). To estimate ΔΨm, cells were transfected with pEGFP‐mito (Clontech) using Lipofectamine 3000 and incubated with 25 nm TMRM for 20 min. ΔΨm is defined as the ratio between TMRM/mito‐GFP (ΔΨm‐sensitive/ΔΨm‐insensitive). To assess cellular ROS levels, cells were incubated with 5 µµ CellROX Deep Red for 15 min.

### 4.13 Mitophagy Detection in hNSCs

Normal hNSCs were seeded on a µ‐Slide 4 Well (Ibidi) and cultured overnight at 37°C. The cells were then treated with 10 µg/mL FCCP for 24 h and co‐stained with mitophagy dye and lysosome dye using a Mitophagy Detection Kit (Dojindo) according to the manufacturer's instructions. Mitochondria were labeled with 500 µm MitoTracker Deep Red. Images were captured using a Leica TCS SP8 confocal microscope (Leica Microsystems, Germany). Quantitative measurements of immunofluorescence images were performed with ImageJ software (ImageJ 1.52a, United States).

### 4.14 Thermal Shift Assay

Cell lysate (2 mg/mL, 50 µL) were prepared from HEK293T cells, and treated with αA (100 µm from a 100× stock in 2‐propanol) or isopropanol for 30 min at room temperature. Each group was then heated for 4 min at different temperatures (35, 41, 47, 53, 59, 65, and 71°C) and subsequently centrifuged for 15 min at 15,000 rpm to separate the supernatant and pellet. Following centrifugation, 12 µL of the supernatant was mixed with 3 µL of 5x loading buffer, and a Western blot experiment was conducted thereafter.

### 4.15 Preparation of Protein Samples

HEK 293 P301S Tau‐Venus cells (100 000 cells/ mL/ well) were seeded in PDL‐coated 6‐well plates using Opti‐MEM and incubated for 24 h in the humidified incubator at 37°C with 5% CO2. To promote endogenous Tau aggregation, 500 µL of Opti‐MEM containing Tau assemblies (20 nM) and 1% Lipofectamine was prepared and added to each well and waited for 90 min in a 37°C incubator. DMEM containing 20% FBS and 1% P&S (total volume 500 µL) was added to each well to allow the formation of endogenous Tau aggregates. After 48 h, the medium was replaced with DMEM (plus 10% FBS and 1% P&S) containing 2‐propanol (Vehicle) or αA (1 µµ) for 48 h. Proteins from each group were collected using 1x RIPA buffer with protease and phosphatase inhibitors.

Frozen hippocampal tissue from WT and AD hTau[P301S] mice after treatment period (vehicle, αA (1 mg/kg, 10 mg/kg, 2 months) were homogenized in a NextAdvance tissue homogenizer with 0.5 mm zirconium oxide beads with 1x RIPA buffer containing protease and phosphatase inhibitors.

Frozen human post‐mortem brain tissue (50 mg) was homogenized in a NextAdvance tissue homogenizer with 0.5 mm zirconium oxide beads in ice‐cold Triton X‐100 buffer containing protease and phosphatase inhibitors (350 µL) at maximum speed for 5 min at 4°C. The supernatant was collected by centrifugation (21 000 g, 15 min) at 4°C. The insoluble pellet was washed 3× in ice‐cold Triton X‐100 buffer and extracted in 1x RIPA buffer containing 8 m Urea, 5% SDS, 1× protease and phosphatase inhibitor.

### 4.16 Western Blot

Protein concentrations were measured using the Pierce BCA Protein Assay Kit. Extracts from cells (15 µg), mouse tissues (30 µg) and postmortem human brain tissue (30 µg) were mixed with 4x sample buffer (0.06 m Tris, 2% SDS, 10% Glycerol, 5% 2‐mercapto‐ethanol) and loaded onto Criterion TGX Precast Stain‐free Gels, or 10–12.5% SDS‐polyacrylamide gels. Gels were transferred to 0.2 µm PVDF membranes, membranes were blocked in 5% BSA for 1 h, probed with primary antibodies overnight at 4°C, washed 3x for 10 min in PBS‐T and incubated for 1 h with 1/5000 anti‐rabbit or anti‐mouse HRP‐conjugated secondary antibodies in PBS‐T. ECL detection system (Pierce) was used, signals were developed using a Curix 60 developer and quantified by densitometry and Fiji/ImageJ.

### 4.17 Animal Strains and Procedures

All procedures in Spain followed EU guidelines for research use of animals and were authorized by CSIC under PROEX244/17. The mito‐QC mitophagy reporter mice in C57BL6/J background were generated and kindly provided by Dr Ian Ganley [63] and were housed at the CIB‐CSIC Animal Facility. Food and water were provided ad libitum and mice were maintained in a 12 h/12 h light/dark cycle. Mice of both sexes were injected intraperitoneally with 1 or 10 mg/kg αA formulated in 30%PEG‐400, 5%Glucose, 5%TWEEN 80, 0.9% NaCl. Mice were anesthetized and immediately perfused with PBS pH 7.0, tissues were collected and fixed by immersion in 3.7% PFA (15710, EMS) with 200 mM HEPES, pH 7.0 for 4 h in an orbiting shaker.

All mouse procedures in China were approved by the Committee on the Ethical Use of Animals in Research at Jinan University and were authorized under project license IACUC‐20230307‐04. hTau[P301S] transgenic AD mice were generated and provided as a gift from Dr. Michel Goedert (Cambridge, UK), and were housed at Jinan University Animal Facility. Food and water were provided ad libitum and mice were maintained in a 12/12 h light/dark cycle. Male and female 9.5‐month‐old mice were treated with αA (1 mg/kg or 10 mg/kg) via oral gavage for 2 months. Tissue samples were obtained immediately after anesthetization.

All procedures performed in Finland were conducted under project license ESAVI/20471/27/9/2019, which was approved by the National Animal Experiment Board of Finland under Directive 2010/63/EU of the European Parliament and the Council of 22 September 2010 on the Protection of Animals Used for Scientific Purposes, with the following provisions: Act (497/2013) and Decree (564/2013) on the Protection of Animals Used for Scientific or Educational Purposes. CD‐1 male mice (6.5 weeks old) were purchased from a commercial supplier (Charles River Laboratories, Germany) and housed in individually ventilated (IVC)‐cages in groups of 3 to 6. The cages were provided with Aspen bedding (4HP and PM90L, Tapvei, Estonia) and paper strands (Sizzlenest, Datesand, UK / EnviroPak with Sizzlenest, Datesand / Bed r'nest, Datesand) as nesting material, and a paper pulp cabin, red polycarbonate cylinder (Datesand, UK) or paper play tunnel (Datesand) and Aspen bricks (S/M‐bricks, Tapvei, Estonia) as cage enrichment. The temperature (22 ± 2°C), humidity (55 ± 10%) and air exchange rate (75 times/hour) of the IVC‐cages and 12/12 h light/dark cycle (500 lux lighting on at 6 a.m., 1.5 lux lighting on at 6 p.m.) of the animal holding room were automatically controlled and maintained. Animals were acclimated for at least 5 days prior to experimental use. Animals were fed ad libitum (Rat/Mouse‐Maintenance extrudate, V1536‐000, ssniff Spezialdiäten GmbH, Germany), had access to tap water at all times, and were observed daily.

### 4.18 mito‐QC Mouse Tissue Processing and Immunofluorescence Imaging

Tissue was embedded in OCT and cryosectioned (CM1900, Leica) to obtain 40‐µm brain sections. For *mito*‐QC analysis, nuclei were counterstained with 1 µg/µL DAPI in PBS pH 7.0. Brain section immunostaining was performed in free‐floating sections in a 24‐well plate as previously described [67]. Briefly, sections were permeabilized with 0.3% Triton X‐100 for 15 min, changed every 5 min, then blocked with block/perm buffer (10% NGS, 0.3% Triton X‐100 in PBS pH 7.0) for 1 h with agitation. Primary antibodies were diluted in block/perm buffer and incubated overnight at the following concentrations: 1/1000 anti‐GFAP, 1/500 anti‐Iba1, 1/200 phospho‐Ubiquitin (Ser65). Sections were washed 3x for 10 min in 0.3% Triton X‐100 in PBS pH 7.0, followed by incubation with secondary antibody or probes diluted in block/perm buffer at the following concentrations: 1/500 anti‐rabbit‐Alexa 647, 1/100 NeuroTrace 640/660, and 1 µg/µL DAPI. Sections were washed three times for 10 min in 0.3% Triton X‐100 and mounted using Vectashield antifade mounting medium. Samples were imaged under a Leica SP8 confocal microscope with 63x immersion objective, and z‐stacks (z‐step 0.5 µm). Mitolysosome quantification was performed in the whole stack using an in‐house Fiji script (ImageJ 1.8.0, Java). Briefly, images were processed to eliminate background using predetermined masks/thresholds for mCherry^+^ and GFP^+^ mitochondria. mCherry^+^ GFP^−^ mitolysosome mask was obtained by subtracting GFP^+^ area from mCherry^+^ mass and quantification was performed using 3D Objects Counter [68]. For mitophagy quantification within cell types, a 3D cell skeleton was reconstructed using a cytoplasmic cell marker (NeuroTrace, neurons; GFAP, astrocytes; Iba1, microglia) and mitolysosomal signal was quantified as previously described. For phospho‐Ubiquitin (Ser65) staining, mean fluorescence intensity (MFI) was measured and co‐localization with mCherry^+^ GFP^+^ and mCherry^+^ GFP^−^ was assessed using JaCoP [68].

### 4.19 Behavioral Studies in Wild Type and hTau[P301S] AD‐Like Mice

Wild type (WT) mice (8 male, 6 female) and hTau[P301S] heterozygous mice were used in behavioral studies. hTau[P301S] AD‐like mice were treated with vehicle (5 male, 4 female), αA (1 mg/kg) (5 male, 4 female), or αA (10 mg/kg) (7 male, 5 female) via oral gavage for two consecutive months starting at 9.5‐months. Morris water maze (MWM), Y‐maze, and novel object recognition (NOR) performance was assessed in hTau[P301S] AD‐like mice after 2 months treatment with αA or control as indicated.

MWM test was performed to assess the learning and spatial memory of mice [69]. A circular white pool (120 cm diameter × 50 cm depth), divided into four equal areas, was filled with TiO2 dyed milky water at 22°C, and a platform (10 cm in diameter) was hidden at a position 1.5 cm under the water surface in the third quadrant. During the training period, mice were trained 3 times per day for 7 consecutive days, and each trial lasted 1 min; after completion, mice were allowed to stay on the platform for another 15 s. If mice were unable to find the platform, the exposure time was counted as 1 min, and the mouse was guided onto the platform where it would stay for 15 s. Assessments were performed on the eighth day. During the assessments, the hidden platform was removed, and mice were placed into the opposite quadrant of the original platform. Within 1 min, the swimming speed, tracks, and swimming time to arrive the quadrant with original platform were automatically recorded using the Labmaze video tracking system (Labmaze V3.0).

Y‐maze test was performed to evaluate the short‐term spatial learning ability in mice. A ‘Y’ shaped white plastic maze with 3 arms (30 cm length × 6 cm width × 20 cm height) set at 120° from each other was used. Mice were placed at the center of the maze and given 10 min to freely explore the maze. The maze was cleaned with 75% ethanol after each trial. The number of entries to each arm was recorded using the Labmaze video tracking system (Labmaze V3.0). The spontaneous alternation = number of times a different arm was entered (alternations) / (total arm entries – 2). The higher percentage of spontaneous changes indicates the better cognitive function.

NOR test [70] was employed to investigate the short‐term cognitive function in mice. A gray plastic box (35 cm length × 35 cm width × 25 cm height) was used as the experimental arena. Before the training time, mice were placed into the experimental box to freely explore the arena environment for 10 min per day for 3 consecutive days. During the training period, individual mice were placed in the same box to explore 3 identical objects for 5 min. After 24 h, mice were placed back into the box with two familiar object and one novel object and the time the mouse spent exploring the new object recorded. The experimental box and objects were cleaned with 75% ethanol after each trial and exploratory behavior was recorded using the Labmaze video tracking system (Labmaze V3.0). The recognition index = time spent on novel object exploration / total object exploration time.

### 4.20 RNA‐Sequencing (RNA‐Seq)

WT mice and hTau[P301S] AD‐like mice were compared after treatment with vehicle or αA (1 or 10 mg/kg). Briefly, hippocampal tissue was collected from anesthetized mice and frozen on dry ice. RNA was isolated using TRIzol reagent, quantified by absorbance 260 nm, and RNA‐seq libraries were prepared using the TruSeq Stranded Total RNA Library Prep Kit (Illumina). Paired‐end (150‐bp reads) sequencing of the RNA libraries was performed on the Illumina Nova6000 platform by ChengQi in China.

### 4.21 RNA‐Seq Data Analysis

Raw fastq from Illumina sequencing data were filtered by Trim galore (v0.6.7), including adapter removal and quality control, to obtain clean fastqs. Reference genome and annotation files were downloaded from https://www.gencodegenes.org/mouse/release_M10.html. The HISAT2 Indexes of the reference genome were constructed, and paired‐end clean fastq reads were aligned to the reference genome with HISAT2 (v2.0.5). FeatureCounts (1.5.0‐p3) was used to map count reads to each gene. TPM values for each gene were calculated. Differential expression was evaluated using pairwise combinations (three biological replicates per group) with DESeq2 R package (v1.36.0). P‐values were adjusted using the Benjamini and Hochberg method to control for the false discovery rate. Cut‐off criteria for DEGs were adjusted *p*‐value <0.05 and log(foldchange) >0.5. Mfuzzy (v2.56.0). R was used to cluster DEGs. GO analysis used the clusterProfiler (v4.4.4) R package. Principal component analysis using factoextra (v1.0.7) and plotly (v 4.1.0). Heatmaps were drawn with pheatmap (v1.0.12). Other data visualizations used ggplot2 (v 3.3.6).

### 4.22 Electron Microscopy

EM analysis of the ultrastructure of mitochondria was described previously [22]. Briefly, hippocampal tissue was collected from anesthetized mice (untreated WT mice, and untreated, 1 or 10 mg/kg of αA‐treated hTau[P301S] AD‐like mice; *n* = 3 per group), and incubated in 2.5% glutaraldehyde (pH 7.4) for 2 h. Tissue samples were washed 3× in 0.1 m phosphate (pH 7.2), and fixed in 1% osmic acid at 4°C for 2 h, dehydrated and embedded in Epon‐Araldite resin, ultra‐thin sectioned, counter‐stained with 3% uranyl acetate and 2.7% lead citrate, and imaged using an HT7800 transmission electron microscope. Ten images per mouse were recorded. For data quantification, the total number of mitochondria and damaged mitochondria were counted. The morphologically changed mitochondria, including loss of mitochondrial cristae and/or membrane, were counted as damaged mitochondria. The percentage of damaged mitochondria = damaged mitochondria / total mitochondria.

### 4.23 Preparation and Analysis of 3D Human AD‐Like Microfluidic Platform

Chemotactic chip design and preparation of 3D human microfluidic platform followed our previous protocol [43, 71]. Briefly, 10 µL AD or control hNPCs were added to the central compartment at 2.5 × 10^6^ cells/mL in culture media/Matrigel at 1:5 (v/v) and 100 µL culture media lacking EGF and bFGF was added to the central chamber and two annular chambers. The microfluidic devices were placed in a 5% CO_2_ cell culture incubator at 37°C. One‐half volume of the media in the central chamber was exchanged with fresh media every 3.5 days for 3 weeks until hNPCs differentiated into neurons and astrocytes. Cultures were treated with vehicle (2‐propanol) or αA (5 µµ) for 6 weeks and culture medium was changed every 3.5 days. At week 9, human adult microglia (10,000 cells per device) were added to the annular chamber in culture media with 2% FBS with same medium added to the central chamber. Cultures were incubated at 37°C for 2 days. Microglia in the central chamber were counted for 2 days using a fully automated Nikon TiE microscope (Nikon, Melville, NY).

### 4.24 Immunocytochemistry

3D AD and control models were rinsed with PBS twice and fixed with 4% paraformaldehyde (PFA, Electron Microscopy Sciences, Hatfield, PA) for 30 min at RT. The models were then rinsed with PBS two times with 10 min intervals and incubated in the permeabilizing solution, PBS solution supplemented with 0.1% (v/v) Triton X‐100 and 0.1% (v/v) Tween 20 (PBSTT), for 30 min at RT. Cells were next washed with PBS three times with 10 min intervals and incubated in the blocking solution, PBS solution supplemented with 0.1% (v/v) Tween 20 and 3% (v/v) human serum albumin (BSA), for 2 h at RT. Cells were again washed with PBS three times with 10 min intervals and incubated with the primary antibody diluted in the blocking solution in 1:100 ratio (v/v). After secondary antibody reaction, the devices were washed seven times with PBS supplemented with 0.1% (v/v) Tween 20 (PBST) with 10 min intervals and examined under a fluorescence microscope (Nikon TiE microscope, Nikon). The intensity of immunoreactivity was analyzed by using a NIS‐Elements software.

### 4.25 Pharmacokinetic Analysis

In vivo pharmacokinetic analysis was performed by Admescope (Symeres, Finland). The formulation of αA for IV was 5% ethanol in 95% clinoleic reagent, prepared no more than 5 h prior to dosing at 5 mg/mL and administered as a single IV dose (10 mg/kg). The animals were weighed and the tail of each animal was marked with an identification number using permanent marker before dosing. The time of dosing and time of first blood sampling were recorded. Blood samples were taken via the saphenous vein (sv) or vena cava (vc) at selected time points (0 mins, 5 mins, 30 mins, and 2, 4, 8, 24, and 48 h). Terminal samples were collected under isoflurane anesthesia. The animals were anesthetized in a chamber infused with oxygen and isoflurane 4% at 1 L/min. Blood samples were collected from the vena cava of anesthetized animals. Within 30 min, blood samples were centrifuged for 10 min, 2700 x g at room temperature, transferred into pre‐labelled plastic tubes and stored at −20°C until analysis. Brain samples were collected 0, 2, 8, 24, and 48 h after dosing. For perfusion, the heart was exposed and major veins leading to the right atrium were severed. A blunt needle was then inserted into the left ventricle of the heart and approximately 10 mL of refrigerated saline was injected to clear remaining blood. Tissue samples were then immediately collected, frozen on dry ice and stored at −20°C until analysis.

Brain samples were weighed and homogenized in PBS (1 mg tissue in 4 µL reagent) using Omni Bead Ruptor 24 homogenizer. Brain homogenate and plasma were precipitated using 4 volumes of ACN with internal standards (100 ng/mL of 5α‐Androstan‐17β‐ol‐3‐one and Testosterone), mixed, and centrifuged for 20 min at 2272 × g at 22°C. Supernatant (150 µL) from each sample was taken and dried in speed‐vac at 40°C and then re‐suspended in 100 µL acetonitrile. Fifty microliters brain homogenate and 30 µL plasma were analyzed by LC‐MS/MS. Standard samples were prepared by spiking blank mouse plasma or blank mouse brain homogenate with 1 to 10 000 ng/mL of the analyte. Quality control (QC) samples were prepared by spiking blank mouse plasma or blank mouse brain homogenate with 3, 30, 300, or 3000 ng/mL of the analyte in duplicate. αA was used as positive control, and p‐toluenesulfonyl isocyanate (PTSI) was used to derivatize αA in the samples to improve αA ionization and sensitivity during electrospray MS. In addition, 20 µL 60% PTSI in acetonitrile was added and derivatization reaction conducted for 3 min with shaking at 1000 rpm at 22°C. The reaction was stopped with 180 µL 5 mm ammonium acetate. A 160 µL aliquot was transferred to a 96‐well plate and 40 µL DMSO was added. The plate was incubated for 1 min on a tabletop shaker prior to LC‐MS/MS analysis. The results were calculated as follows:

$$S=\sqrt{\sum \begin{array}{c} D^{2}\\ 2\left(N-1\right) \end{array}}$$

where *D* ^2^ =${\left(100*\;(\frac{x_{1}-x_{2}}{\bar{x}})\right)}^{2}\;$, *x*_1_ = concentration of standard sample replicate 1, *x*_2 _= concentration of standard sample replicate 2, $\bar{x}$ = average of replicates, and *N* = number of standard concentrations.

### 4.26 Kinase Screening by KINOMEscan

Kinase assays (KINOME*scan*) were performed by Eurofins DiscoverX Corporation. Briefly, the kinases were prepared from kinase‐tagged T7 phage strains and HEK‐293 cell lysates and subsequently tagged with DNA for qPCR detection. The streptavidin‐coated magnetic beads were treated with biotinylated small molecule ligands for 30 min at room temperature to generate affinity resins for kinase assays. The liganded beads were washed with blocking buffer (SeaBlock (Pierce), 1% BSA, 0.05% Tween 20, 1 mM DTT) to remove unbound ligand and to reduce non‐specific phage binding, blocked with excess biotin, and treated with αA (1 µµ). Assays (20 µL) were performed in polypropylene 384‐well plates at room temperature for 1 h with shaking. Affinity beads were washed once in 1x PBS, 0.05%, Tween 20), re‐suspended in elution buffer (1x PBS, 0.05% Tween 20, 0.5 µm non‐biotinylated affinity ligand) and incubated at room temperature with shaking for 30 min. Kinase concentration was measured by qPCR. The primary screen binding interactions are reported as ‘percent control (% Ctrl)’, where lower numbers indicate stronger hits in the matrix. The results were calculated as follows:

$$\%\;Ctrl=\frac{\mathrm{test}\;\mathrm{compound}\;\mathrm{signal}-\mathrm{positive}\;\mathrm{control}\;\mathrm{signal}}{\mathrm{negative}\;\mathrm{control}\;\mathrm{signal}-\mathrm{positive}\;\mathrm{control}\;\mathrm{signal}}\times 100$$

The top hits were identified using % *Ctrl* calculated selective score (S‐score). In the present study, S(35) = (number of non‐mutant kinases with %Ctrl <35)/(number of non‐mutant kinases tested) was used as a potency threshold.

### 4.27 Protein and Ligand Preparation and Induced‐Fit Docking

The crystal structure of human DLK structure (PDB ID: 8OUT and 8OUR) was optimized using the Protein Preparation Wizard in Schrödinger's Maestro (Schrödinger, 2023) and adding bond orders and hydrogen atoms to the crystal structure using the OPLS4 force field. Prime was used to fix missing residues or atoms in the protein and to remove co‐crystallized water molecules. PROPKA was used to check for the protonation state of ionizable protein groups (pH = 7.0). The hydrogen bonds were optimized through the reorientation of hydroxyl bonds, thiol groups, and amide groups. Finally, the system was minimized setting the value of convergence of the RMSD at 0.3 Å. The ligands were prepared using LigPrep. The force field adopted was OPLS4 [72] and Epik 3.9 (Schrödinger, 2023–4) was selected as an ionization tool at pH 7.2 ± 0.2. Tautomer generation was unflagged and the maximum number of conformers generated was 32.

Induced‐fit docking is a method for modeling conformational changes induced by ligand binding [73]. This protocol models induced‐fit docking of one or more ligands as previously reported [74] using softened potential (van der Waals radii scaling) followed by a side‐chain prediction within a specified distance of any ligand. Subsequently, a minimization of the same set of residues and the ligand for each protein/ligand complex pose is performed. After this stage, any receptor structure in each pose reflects an induced fit to the ligand structure and conformation. Finally, the ligand is rigorously docked, using Glide XP, into the induced‐fit receptor structure. The grid boxes for the binding sites were based on the co‐crystallized ligand W3C (8OUT) and W38 (8OUR) as a centroid. During the initial docking procedure, the van der Waals scaling factor was set at 0.5 for both receptor and ligand. The Prime refinement step was set on side chains of residues within 5 Å of the ligand. For each ligand docked, a maximum of 20 poses was retained to be redocked using XP mode.

### 4.28 Statistical Analysis

The Kaplan–Meier curve with Log‐rank test was performed for the cumulative incidence rate of dementia by follow‐up time. Pearson's correlation coefficient r was used to evaluate the relationship between the between the AFVC and plasma p‐Tau217 level at baseline. Tests were set at a significance level of 0.05 with two tails. In vitro and in vivo experiments were performed with at least three biological repeats unless otherwise noted. Imaging data were quantified using ImageJ software. Statistical analysis was performed with GraphPad Prism 8.0 and Prism 10.4.1. Unless otherwise noted, all data are presented as mean ± SEM, and values of *p* < 0.05 were considered significant. Two‐tailed unpaired Student's t‐tests was used when comparing the means of two groups; the dependent variable was continuous and the data were approximately normally distributed. One‐way analysis of variance (ANOVA) followed by Dunnett's multiple comparisons test was employed to compare multiple groups sharing a single categorical independent variable; two‐way ANOVA followed by Tukey's or Dunnett's multiple comparisons tests were used to test the effects of two independent variables (factors) on a dependent variable.

## Author Contributions

E.F.F., T.T., and P.B. designed the experiments with E.F.F. provided overall scientific guidance. S.Q.C., J.I.J.‐L., Y.G.Q., J.J.H., Y.J.K., K.D.A., A.E.S., J.P.P, L.P.M., A.L., H.G.Y., and A.B.C. performed the experiments. Y.A., M.J.D.L., S. L., K.X.L., T.C.C., H.L.Z., J.Y., X.R.J., O.A., F.P., R.M., W.A.M., F.X.C, H.S.C., G.B.C., H.X.S., K.P., O.J.L., N.T., and C.V.D. provided experimental materials. Q.H.Z., and D.D. provided the community cohort data. S.Q.C., J.I.J.‐L., P.B., T.T., and E.F.F. wrote the manuscript. All authors read and commented on the manuscript.

## Funding

The authors confirm this project was funded by the Molecule AG/VITADAO (#282942), the Research Council of Norway (#262175 and #334361), HELSE SØR‐ØST (#2020001, #2021021, and #2023093), NordForsk Foundation (#119986), the National Natural Science Foundation of China (#81971327), Akershus University Hospital (#269901, #261973, and #262960), the Civitan Norges Forskningsfond for Alzheimers sykdom (#281931), the Czech Republic‐Norway KAPPA programme (with Martin Vyhnálek, #TO01000215), HORIZON‐TMA‐MSCA‐DN (#101073251, with Riekelt Houtkooper), and Wellcome Leap's Dynamic Resilience Program (jointly funded by Temasek Trust) (#104617). S.Q.C. is supported by Nasjonalforeningen for folkehelsen (#43622), the scholarships from the Graduate School, Chulalongkorn University to commemorate 72^nd^ Anniversary of his Majesty King Bhumibol Adulyadej and the 100^th^ Anniversary Chulalongkorn University Fund for Doctoral Scholarship as well as the 90^th^ Anniversary Chulalongkorn University Fund (Ratchadaphiseksomphot Endowment Fund) and Overseas Research Experience scholarship for Graduate students. J. I. J.‐L. is supported by an FPI fellowship from MCIN (PRE2019‐088222). G.C. is supported by Guangdong Basic and Applied Basic Research Foundation (2022B1515120043). J.P.P is funded by the Natural Science Foundation of China (grant no. 82301422). K.P. is supported by grants from the Fondation Santé (19656) and the European Research Council (GA 101077374 – SynaptoMitophagy). N.T. is supported by the European Research Council, under the grant agreement ERC‐GA695190‐MANNA. H.C. and K.V.D are supported by the National Research Foundation (NRF‐2020R1A2C2010285 and NRF‐I21SS7606036), the Ministry of Health & Welfare and Ministry of Science and ICT (HU22C0115). R.M is funded by the Hellenic Fundation for Research and Innovation grant HFRI‐1019 DiseasePhenoTarget. Y.J.K. is supported by NRF‐2022R1I1A1A01063094. Q.H.Z. is supported by National Natural Science Foundation of China (# 82371429), D.D. is supported by the National Natural Science Foundation of China (#82473701 and #82173599). Research in P.B. lab is supported by grant PID2021‐126864NB‐I00 from MCIN, Spain, the University of Fribourg and grant 310030‐215271 from the SNSF, Switzerland.

## Ethics Statement

The Shanghai Aging Study was approved by the Medical Ethics Committee of Huashan Hospital, Fudan University (No. 2009–195). The iPSC was approved by the Western Norway Committee for Ethics in Health Research (REK2012/919). All human postmortem hippocampal tissue were collected From King's College London, UK Under the regional Ethical approval From medical research in the South‐East of Norway (REK 82685) and the Data Protector Officer, Norway. The mouse experiments were approved by the regional animal ethics committee (FOTS ID 29730)

## Consent

All participants and/or their legal representatives signed the written informed consent.

## Conflicts of Interest

E.F.F. is a co‐owner of Fang‐S Consultation AS (Organization number 931 410 717) and NO‐Age AS (Organization number 933 219 127); he has an MTA with LMITO Therapeutics Inc (South Korea), and a CRADA arrangement with ChromaDex (USA); he is a consultant to MindRank AI (China), NYO3 (Norway), and AgeLab (Vitality Nordic AS, Norway). E.F.F. and S.Q.C. have a commercialization agreement with Molecule AG/VITADAO. T.C.C. is a Consultant of Nin Jiom Medicine Manufactory (H.K) Ltd and HK Longevity Sciences Laboratory Limited. The other authors have declared no competing interests.

## Supporting information




**Supporting File**: advs73924‐sup‐0001‐SuppMat.docx.

## Data Availability

All data are in the manuscript and the associated supporting information file. Additional raw data are available per reasonable request to E.F.F.

## References

[1] E. F. Fang , M. Scheibye‐Knudsen , H. J. Jahn , et al., “A Research Agenda for Aging in China in the 21st Century,” Ageing Research Reviews 24 (2015): 197–205. 26304837 10.1016/j.arr.2015.08.003 PMC5179143

[2] E. F. Fang , Y. Fang , G. Chen , H.‐L. Wang , and J. Zhang , “Adapting Health, Economic and Social Policies to Address Population Aging in China,” Nature Aging (2025): 1–12. 41174222 10.1038/s43587-025-00999-8

[3] A.‐J. Tessier , F. Wang , A. A. Korat , et al., “Optimal Dietary Patterns for Healthy Aging,” Nature Medicine 31 (2025): 1644–1652, 10.1038/s41591-025-03570-5. PMC12092270 40128348

[4] T. Ballarini , D. M. van Lent , J. Brunner , et al., “Mediterranean Diet, Alzheimer Disease Biomarkers, and Brain Atrophy in Old Age,” Neurology 96 (2021): e2920–e2932. 33952652 10.1212/WNL.0000000000012067 PMC8253566

[5] P. Agarwal , S. E. Leurgans , S. Agrawal , et al., “Association of Mediterranean‐DASH Intervention for Neurodegenerative Delay and Mediterranean Diets with Alzheimer Disease Pathology,” Neurology 100 (2023): e2259–e2268, 10.1212/WNL.0000000000207176. 36889921 PMC10259273

[6] A. M. Brickman , L.‐K. Yeung , D. M. Alschuler , et al., “Dietary Flavanols Restore Hippocampal‐Dependent Memory in Older Adults with Lower Diet Quality and Lower Habitual Flavanol Consumption,” Proceedings of the National Academy of Sciences 120 (2023): 2216932120, 10.1073/pnas.2216932120. PMC10265949 37252983

[7] A. Dement , “Alzheimer's Disease Facts and Figures,” Alzheimer's & Dementia: The Journal of the Alzheimer's Association 12 (2016): 459–509. 10.1016/j.jalz.2016.03.001 27570871

[8] R. G. Canter , J. Penney , and L. H. Tsai , “The Road to Restoring Neural Circuits for the Treatment of Alzheimer's Disease,” Nature 539 (2016): 187–196, 10.1038/nature20412. 27830780

[9] J. S. Kerr , B. A. Adriaanse , N. H. Greig , et al., “Mitophagy and Alzheimer's Disease: Cellular and Molecular Mechanisms,” Trends in Neurosciences 40 (2017): 151–166, 10.1016/j.tins.2017.01.002. 28190529 PMC5341618

[10] M. N. Goedert , “Alzheimer's and Parkinson's Diseases: The Prion Concept in Relation to Assembled Aβ, tau, and α‐Synuclein,” Science 349 (2015): 1255555, 10.1126/science.1255555. 26250687

[11] G. V. Johnson and W. H. Stoothoff , “Tau Phosphorylation in Neuronal Cell Function and Dysfunction,” Journal of Cell Science 117 (2004): 5721–5729, 10.1242/jcs.01558. 15537830

[12] T. Rodríguez‐Martín , I. Cuchillo‐Ibáñez , W. Noble , F. Nyenya , B. H. Anderton , and D. P. Hanger , “Tau Phosphorylation Affects Its Axonal Transport and Degradation,” Neurobiology of Aging 34 (2013): 2146–2157, 10.1016/j.neurobiolaging.2013.03.015. 23601672 PMC3684773

[13] M. Goedert , “Tau Proteinopathies and the Prion Concept,” Progress in Molecular Biology and Translational Science 175 (2020): 239–259. 32958235 10.1016/bs.pmbts.2020.08.003

[14] N. J. Ashton , S. Janelidze , N. Mattsson‐Carlgren , et al., “Differential Roles of Aβ42/40, p‐tau231 and p‐tau217 for Alzheimer's Trial Selection and Disease Monitoring,” Nature Medicine 28 (2022): 2555–2562, 10.1038/s41591-022-02074-w. PMC9800279 36456833

[15] N. J. Ashton , W. S. Brum , G. Di Molfetta , et al., “Diagnostic Accuracy of a Plasma Phosphorylated Tau 217 Immunoassay for Alzheimer Disease Pathology,” JAMA Neurology 81 (2024): 255–263, 10.1001/jamaneurol.2023.5319. 38252443 PMC10804282

[16] Z. Xiao , W. Wu , X. Ma , et al., “Plasma p‐tau217, p‐tau181, and NfL as Early Indicators of Dementia Risk in a Community Cohort: The Shanghai Aging Study,” Alzheimer's & Dementia: Diagnosis, Assessment & Disease Monitoring 15 (2023): 12514. 10.1002/dad2.12514 PMC10740382 38145191

[17] M. M. Mielke , J. L. Dage , R. D. Frank , et al., “Performance of Plasma Phosphorylated Tau 181 and 217 in the Community,” Nature Medicine 28 (2022): 1398–1405, 10.1038/s41591-022-01822-2. PMC9329262 35618838

[18] R. H. Swerdlow , “Mitochondria and Cell Bioenergetics: Increasingly Recognized Components and a Possible Etiologic Cause of Alzheimer's Disease,” Antioxidants & Redox Signaling 16 (2012): 1434–1455, 10.1089/ars.2011.4149. 21902597 PMC3329949

[19] T. Ashleigh , R. H. Swerdlow , and M. F. Beal , “The Role of Mitochondrial Dysfunction in Alzheimer's Disease Pathogenesis,” Alzheimer's & Dementia 19 (2023): 333–342, 10.1002/alz.12683. 35522844

[20] Y. Aman , T. Schmauck‐Medina , M. Hansen , et al., “Autophagy in Healthy Aging and Disease,” Nature Aging 1 (2021): 634–650, 10.1038/s43587-021-00098-4. 34901876 PMC8659158

[21] S.‐Q. Cao , Y. Aman , E. F. Fang , and T. P. Tencomnao , “ *P. edulis* Extract Protects Against Amyloid‐β Toxicity in Alzheimer's Disease Models Through Maintenance of Mitochondrial Homeostasis via the FOXO3/DAF‐16 Pathway,” Molecular Neurobiology 59 (2022): 5612–5629, 10.1007/s12035-022-02904-5. 35739408

[22] E. F. Fang , Y. Hou , K. Palikaras , et al., “Mitophagy Inhibits Amyloid‐β and Tau Pathology and Reverses Cognitive Deficits in Models of Alzheimer's Disease,” Nature Neuroscience 22 (2019): 401–412, 10.1038/s41593-018-0332-9. 30742114 PMC6693625

[23] C. Xie , X.‐X. Zhuang , Z. Niu , et al., “Amelioration of Alzheimer's Disease Pathology by Mitophagy Inducers Identified via Machine Learning and a Cross‐Species Workflow,” Nature Biomedical Engineering 6 (2022): 76–93, 10.1038/s41551-021-00819-5. PMC8782726 34992270

[24] S. Tang , Z. Xiao , F. Lin , et al., “Joint Effect of Testosterone and Neurofilament Light Chain on Cognitive Decline in Men: The Shanghai Aging Study,” Alzheimer's & Dementia 20 (2024): 5290–5298, 10.1002/alz.13889. PMC11350006 38837321

[25] F. Cheng , W. Lu , C. Liu , et al., “A Genome‐wide Positioning Systems Network Algorithm for in Silico Drug Repurposing,” Nature Communications 10 (2019): 3476, 10.1038/s41467-019-10744-6. PMC6677722 31375661

[26] F. Cheng , R. J. Desai , D. E. Handy , et al., “Network‐Based Approach to Prediction and Population‐Based Validation of in Silico Drug Repurposing,” Nature communications 9 (2018): 2691, 10.1038/s41467-018-05116-5. PMC6043492 30002366

[27] J. Fang , P. Zhang , Y. Zhou , et al., “Endophenotype‐Based In Silico Network Medicine Discovery Combined with Insurance Record Data Mining Identifies Sildenafil as a Candidate Drug for Alzheimer's Disease,” Nature Aging 1 (2021): 1175–1188, 10.1038/s43587-021-00138-z. 35572351 PMC9097949

[28] W. A. McEwan , B. Falcon , M. Vaysburd , et al., “Cytosolic Fc Receptor TRIM21 Inhibits Seeded Tau Aggregation,” Proceedings of the National Academy of Sciences 114 (2017): 574–579, 10.1073/pnas.1607215114. PMC5255578 28049840

[29] A. S. Mukadam , L. V. C. Miller , A. E. Smith , et al., “Cytosolic Antibody Receptor TRIM21 Is Required for Effective Tau Immunotherapy in Mouse Models,” Science 379 (2023): 1336–1341, 10.1126/science.abn1366. 36996217 PMC7614512

[30] J. H. Brelstaff , M. Mason , T. Katsinelos , et al., “Microglia Become Hypofunctional and Release Metalloproteases and Tau Seeds When Phagocytosing Live Neurons with P301S Tau Aggregates,” Science Advances 7 (2021): abg4980, 10.1126/sciadv.abg4980. PMC8528424 34669475

[31] B. Allen , E. Ingram , M. Takao , et al., “Abundant Tau filaments and Nonapoptotic Neurodegeneration in Transgenic Mice Expressing human P301S Tau Protein,” Journal of Neuroscience 22 (2002): 9340–9351, 10.1523/JNEUROSCI.22-21-09340.2002. 12417659 PMC6758022

[32] L. E. Jarrard , “On the Role of the Hippocampus in Learning and Memory in the Rat,” Behavioral and Neural Biology 60 (1993): 9–26, 10.1016/0163-1047(93)90664-4. 8216164

[33] J. I. Jiménez‐Loygorri , R. Benítez‐Fernández , Á. Viedma‐Poyatos , et al., “Mitophagy in the Retina: Viewing Mitochondrial Homeostasis Through a New Lens,” Progress in Retinal and Eye Research 96 (2023): 101205, 10.1016/j.preteyeres.2023.101205. 37454969

[34] N. Abolhassani , J. Leon , Z. Sheng , et al., “Molecular Pathophysiology of Impaired Glucose Metabolism, Mitochondrial Dysfunction, and Oxidative DNA Damage in Alzheimer's Disease Brain,” Mechanisms of Ageing and Development 161 (2017): 95–104, 10.1016/j.mad.2016.05.005. 27233446

[35] C. D. Pozniak , A. Sengupta Ghosh , A. Gogineni , et al., “Dual Leucine Zipper Kinase Is Required for Excitotoxicity‐Induced Neuronal Degeneration,” Journal of Experimental Medicine 210 (2013): 2553–2567, 10.1084/jem.20122832. 24166713 PMC3832926

[36] D. W. Summers , E. Frey , L. J. Walker , J. Milbrandt , and A. DiAntonio , “DLK Activation Synergizes with Mitochondrial Dysfunction to Downregulate Axon Survival Factors and Promote SARM1‐Dependent Axon Degeneration,” Molecular Neurobiology 57 (2020): 1146–1158, 10.1007/s12035-019-01796-2. 31696428 PMC7035184

[37] H. M. C. Choi , Y. Li , D. Suraj , et al., “Autophagy Protein ULK1 Interacts with and Regulates SARM1 During Axonal Injury,” Proceedings of the National Academy of Sciences 119 (2022): 2203824119, 10.1073/pnas.2203824119. PMC9704737 36375051

[38] D. F. Egan , D. B. Shackelford , M. M. Mihaylova , et al., “Phosphorylation of ULK1 (hATG1) by AMP‐Activated Protein Kinase Connects Energy Sensing to Mitophagy,” Science 331 (2011): 456–461, 10.1126/science.1196371. 21205641 PMC3030664

[39] E. H. Thijssen , R. La Joie , A. Strom , et al., “Plasma Phosphorylated Tau 217 and Phosphorylated tau 181 as Biomarkers in Alzheimer's Disease and Frontotemporal Lobar Degeneration: A Retrospective Diagnostic Performance Study,” Lancet Neurology 20 (2021): 739–752, 10.1016/S1474-4422(21)00214-3. 34418401 PMC8711249

[40] T. K. Karikari , A. Emersic , A. Vrillon , et al., “Head‐to‐Head Comparison of Clinical Performance of CSF Phospho‐tau T181 and T217 Biomarkers for Alzheimer's Disease Diagnosis,” Alzheimer's & Dementia 17 (2021): 755–767, 10.1002/alz.12236. PMC8246793 33252199

[41] N. R. Barthélemy , Y. Li , N. Joseph‐Mathurin , et al., “A Soluble Phosphorylated Tau Signature Links Tau, Amyloid and the Evolution of Stages of Dominantly Inherited Alzheimer's Disease,” Nature Medicine 26 (2020): 398–407, 10.1038/s41591-020-0781-z. PMC7309367 32161412

[42] H. Braak and E. Braak , “Neuropathological Stageing of Alzheimer‐Related Changes,” Acta Neuropathologica 82 (1991): 239–259, 10.1007/BF00308809. 1759558

[43] Y. J. Kang , Y. N. Diep , M. Tran , et al., “Three‐Dimensional Human Neural Culture on a Chip Recapitulating Neuroinflammation and Neurodegeneration,” Nature Protocols 18 (2023): 2838–2867, 10.1038/s41596-023-00861-4. 37542184

[44] J. Park , I. Wetzel , I. Marriott , et al., “A 3D Human Triculture System Modeling Neurodegeneration and Neuroinflammation in Alzheimer's Disease,” Nature Neuroscience 21 (2018): 941–951, 10.1038/s41593-018-0175-4. 29950669 PMC6800152

[45] Y. H. Kim , S. H. Choi , C. D'Avanzo , et al., “A 3D Human Neural Cell Culture System for Modeling Alzheimer's Disease,” Nature Protocols 10 (2015): 985–1006, 10.1038/nprot.2015.065. 26068894 PMC4499058

[46] P. Williams , A. Sorribas , and M.‐J. R. Howes , “Natural Products as a Source of Alzheimer's Drug Leads,” Natural Product Reports 28 (2011): 48–77, 10.1039/C0NP00027B. 21072430 PMC4917364

[47] A. G. Atanasov , S. B. Zotchev , V. M. Dirsch , and C. T. Supuran , “Natural Products in Drug Discovery: Advances and Opportunities,” Nature Reviews Drug Discovery 20 (2021): 200–216, 10.1038/s41573-020-00114-z. 33510482 PMC7841765

[48] G. Civiletto , D. Brunetti , G. Lizzo , K. Muller , and G. E. Jacot , “Herbal Terpenoids Activate Autophagy and Mitophagy Through Modulation of Bioenergetics and Protect From Metabolic Stress, Sarcopenia and Epigenetic Aging,” Nature Aging 5 (2025): 2003–2021. 40993327 10.1038/s43587-025-00957-4 PMC12532568

[49] I. Gutiérrez‐del‐Río , J. Fernández , and F. Lombó , “Plant Nutraceuticals as Antimicrobial Agents in Food Preservation: Terpenoids, Polyphenols and Thiols,” International Journal of Antimicrobial Agents 52 (2018): 309–315. 29777759 10.1016/j.ijantimicag.2018.04.024

[50] M. F. Gulen , N. Samson , A. Keller , et al., “cGAS–STING Drives Ageing‐Related Inflammation and Neurodegeneration,” Nature 620 (2023): 374–380, 10.1038/s41586-023-06373-1. 37532932 PMC10412454

[51] S. Lautrup , D. A. Sinclair , M. P. Mattson , and E. F. Fang , “NAD^+^ in Brain Aging and Neurodegenerative Disorders,” Cell Metabolism 30 (2019): 630–655, 10.1016/j.cmet.2019.09.001. 31577933 PMC6787556

[52] J. Zhang , H.‐L. Wang , S. Lautrup , H. L. Nilsen , and J. T. Treebak , “Emerging Strategies, Applications and Challenges of Targeting NAD^+^ in the Clinic,” Nature Aging 5 (2025): 1704–1731. 40926126 10.1038/s43587-025-00947-6

[53] M. Siu , A. Sengupta Ghosh , and J. W. Lewcock , “Dual Leucine Zipper Kinase Inhibitors for the Treatment of Neurodegeneration,” Journal of Medicinal Chemistry 61 (2018): 8078–8087, 10.1021/acs.jmedchem.8b00370. 29863360

[54] N. Watamura , M. S. Foiani , S. Bez , M. Bourdenx , and A. Santambrogio , “In Vivo Hyperphosphorylation of Tau Is Associated With Synaptic Loss and Behavioral Abnormalities in the Absence of Tau Seeds,” Nature Neuroscience 28 (2024): 293–307. 39719507 10.1038/s41593-024-01829-7 PMC11802456

[55] S. Patel , W. J. Meilandt , R. I. Erickson , et al., “Selective Inhibitors of Dual Leucine Zipper Kinase (DLK, MAP3K12) with Activity in a Model of Alzheimer's Disease,” Journal of Medicinal Chemistry 60 (2017): 8083–8102, 10.1021/acs.jmedchem.7b00843. 28929759

[56] M. D. Figley , W. Gu , J. D. Nanson , et al., “SARM1 is a Metabolic Sensor Activated by an Increased NMN/NAD^+^ Ratio to Trigger Axon Degeneration,” Neuron 109 (2021): 1118–1136, 10.1016/j.neuron.2021.02.009. 33657413 PMC8174188

[57] B. Wang , R. Iyengar , X. Li‐Harms , et al., “The Autophagy‐inducing Kinases, ULK1 and ULK2, Regulate Axon Guidance in the Developing Mouse Forebrain via a Noncanonical Pathway,” Autophagy 14 (2018): 796–811, 10.1080/15548627.2017.1386820. 29099309 PMC6070005

[58] W. M. Pardridge , “Drug and Gene Targeting to the Brain with Molecular Trojan Horses,” Nature Reviews Drug Discovery 1 (2002): 131–139, 10.1038/nrd725. 12120094

[59] D. A. Smith , K. Beaumont , T. S. Maurer , and L. Di , “Relevance of Half‐Life in Drug Design,” Journal of Medicinal Chemistry 61 (2018): 4273–4282, 10.1021/acs.jmedchem.7b00969. 29112446

[60] S. Liu , J. Luo , Z. Xiao , et al., “Low Dietary Vitamin E Intake Is Associated with High Risk of Incident Dementia Among Older Adults: The Shanghai Aging Study,” Frontiers in Nutrition 9 (2022): 1036795, 10.3389/fnut.2022.1036795. 36505244 PMC9727246

[61] A. Gaulton , L. J. Bellis , and A. P. Bento , “ChEMBL: A Large‐Scale Bioactivity Database for Drug Discovery,” Nucleic Acids Research 40 (2012), D1100–D1107. 21948594 10.1093/nar/gkr777 PMC3245175

[62] T. Liu , Y. Lin , X. Wen , R. N. Jorissen , and M. K. Gilson , “BindingDB: A Web‐Accessible Database of Experimentally Determined Protein–Ligand Binding Affinities,” Nucleic Acids Research 35 (2007), D198–D201. 17145705 10.1093/nar/gkl999 PMC1751547

[63] L. Montava‐Garriga , F. Singh , G. Ball , and I. G. Ganley , “Semi‐Automated Quantitation of Mitophagy in Cells and Tissues,” Mechanisms of Ageing and Development 185 (2020): 111196, 10.1016/j.mad.2019.111196. 31843465 PMC6961211

[64] K. X. Liang , C. K. Kristiansen , S. Mostafavi , et al., “Disease‐Specific Phenotypes in iPSC ‐Derived Neural Stem Cells with *POLG* Mutations,” EMBO Molecular Medicine 12 (2020): 12146, 10.15252/emmm.202012146. PMC7539330 32840960

[65] S. R. L. Stacpoole , B. Bilican , D. J. Webber , et al., “Efficient Derivation of NPCs, Spinal Motor Neurons and Midbrain Dopaminergic Neurons from hESCs at 3% Oxygen,” Nature Protocols 6 (2011): 1229–1240, 10.1038/nprot.2011.380. 21799491 PMC3433269

[66] B. J. Tuck , L. V. C. Miller , T. Katsinelos , et al., “Cholesterol Determines the Cytosolic Entry and Seeded Aggregation of Tau,” Cell Reports 39 (2022): 110776, 10.1016/j.celrep.2022.110776. 35508140 PMC9108550

[67] F. Singh , A. R. Prescott , P. Rosewell , G. Ball , A. D. Reith , and I. G. Ganley , “Pharmacological Rescue of Impaired Mitophagy in Parkinson's Disease‐Related LRRK2 G2019S Knock‐In Mice,” Elife 10 (2021): 67604. 10.7554/eLife.67604 PMC8331189 34340748

[68] S. Bolte and F. P. Cordelières , “A Guided Tour Into Subcellular Colocalization Analysis in Light Microscopy,” Journal of Microscopy 224 (2006): 213–232, 10.1111/j.1365-2818.2006.01706.x. 17210054

[69] C. V. Vorhees and M. T. Williams , “Morris Water Maze: Procedures for Assessing Spatial and Related Forms of Learning and Memory,” Nature Protocols 1 (2006): 848–858, 10.1038/nprot.2006.116. 17406317 PMC2895266

[70] M. Leger , A. Quiedeville , V. Bouet , et al., “Object Recognition Test in Mice,” Nature Protocols 8 (2013): 2531–2537, 10.1038/nprot.2013.155. 24263092

[71] H. Cho , T. Hashimoto , E. Wong , et al., “Microfluidic Chemotaxis Platform for Differentiating the Roles of Soluble and Bound Amyloid‐β on Microglial Accumulation,” Scientific Reports 3 (2013): 1–7, 10.1038/srep01823. PMC3650586 23665843

[72] C. Lu , C. Wu , D. Ghoreishi , et al., “OPLS4: Improving Force Field Accuracy on Challenging Regimes of Chemical Space,” Journal of Chemical Theory and Computation 17 (2021): 4291–4300, 10.1021/acs.jctc.1c00302. 34096718

[73] W. Sherman , T. Day , M. P. Jacobson , R. A. Friesner , and R. Farid , “Novel Procedure for Modeling Ligand/Receptor Induced Fit Effects,” Journal of Medicinal Chemistry 49 (2006): 534–553, 10.1021/jm050540c. 16420040

[74] M. Tutone , I. Pibiri , L. Lentini , A. Pace , and A. M. Almerico , “Deciphering the Nonsense Readthrough Mechanism of Action of Ataluren: An *In Silico* Compared Study,” ACS Medicinal Chemistry Letters 10 (2019): 522–527, 10.1021/acsmedchemlett.8b00558. 30996790 PMC6466511

[75] S. M. Holland , K. M. Collura , A. Ketschek , et al., “Palmitoylation Controls DLK localization, Interactions and Activity to Ensure Effective Axonal Injury Signaling,” Proceedings of the National Academy of Sciences 113, no. 3 (2016): 763–768. 10.1073/pnas.1514123113 PMC4725513 26719418

