# Supplementary material for: The Mitochondrial Guardian α‐Amyrin Mitigates Alzheimer's Disease Pathology via Modulation of the DLK‐SARM1‐ULK1 Axis
Source: Adv Sci (Weinh). 2026 Jan 22;13(24):e12374. doi: 10.1002/advs.202512374 (PMC13116123; doi:10.1002/advs.202512374)
Supplement: Supplementary file 1 — Supporting File: advs73924‐sup‐0001‐SuppMat.docx. [file ADVS-13-e12374-s001.docx]

**Supplementary Table 1 The distribution of top 10 anti-AD hits with Z-score**

| Compound | Source | Z-score |
| --- | --- | --- |
| Apigenin | Tomato | -5.403 |
| Myricetin | \| American cranberry \| \| --- \| \| Bell Pepper \| \| European cranberry \| \| Tomato \| | -5.281 |
| Quercetin | \| American cranberry \| \| --- \| \| Apple \| \| European cranberry \| \| Sweet cherry \| \| Tomato \| | -4.559 |
| Nicotinic acid | \| American cranberry \| \| --- \| \| Apple \| \| Asian pear \| \| Bell Pepper \| \| Eggplant \| \| European Grape \| \| Grape \| \| Passion fruit \| \| Tomato \| | -4.533 |
| protocatechuate | \| Olive \| \| --- \| \| Eggplant \| | -4.512 |
| Isoorientin | Olive | -4.467 |
| Kaempferol | \| American cranberry \| \| --- \| \| Tomato \| | -4.388 |
| alpha-Amyrin | \| American cranberry \| \| --- \| \| Asian pear \| \| Bell Pepper \| \| Eggplant \| \| European cranberry \| \| Grape \| \| Olive \| \| Passion fruit \| \| Sweet cherry \| \| Tomato \| | -4.198 |
| Chlorogenic acid | \| Apple \| \| --- \| \| European cranberry \| \| Olive \| \| Sweet cherry \| \| Tomato \| | -4.112 |
| Luteolin | \| Bell Pepper \| \| --- \| \| Olive \| | -4.07 |

**Supplementary Table 2** **Parameters of Pharmacokinetic analysis of α-Amyrin following intravenous (IV) administration in CD-1 mice.**

| **Parameter** | **Estimate** | **Units** |
| --- | --- | --- |
| T_1/2_ | 10.1 | h |
| C_0_ | 7050 | ng/mL |
| C_max_ | 5930 | ng/mL |
| T_max_ | 0,083 | h |
| C_last_ | 87,4 | ng/mL |
| T_last_ | 48 | h |
| AUC_0-last_ | 13700 | h*ng/mL |
| AUC_0-infinity_ | 15000 | h*ng/mL |
| AUC (% Extrapolated) | 8.52 | % |
| Clearance | 11.1 | mL/min/kg |
| Vss | 9.54 | L/kg |
| Mean (Brain to Plasma Ration, 2 hours) | 0.0212 | % |
| Mean (Brain to Plasma Ration, 8 hours) | 0.0908 | % |

**Supplementary Table 3 Ten complex conformations between α-Amyrin and DLK generate through cross-docking**

| Model | Affinity (kcal/mol) | RMSD |
| --- | --- | --- |
| 1 | -9.878 | 0.0000 |
| 2 | -9.786 | 0.1657 |
| 3 | -9.523 | 0.1229 |
| 4 | -9.264 | 0.1683 |
| 5 | -9.007 | 0.4849 |
| 6 | -8.811 | 0.1762 |
| 7 | -8.688 | 0.2346 |
| 8 | -8.364 | 0.1515 |
| 9 | -8.226 | 0.3261 |
| 10 | -7.973 | 0.3092 |


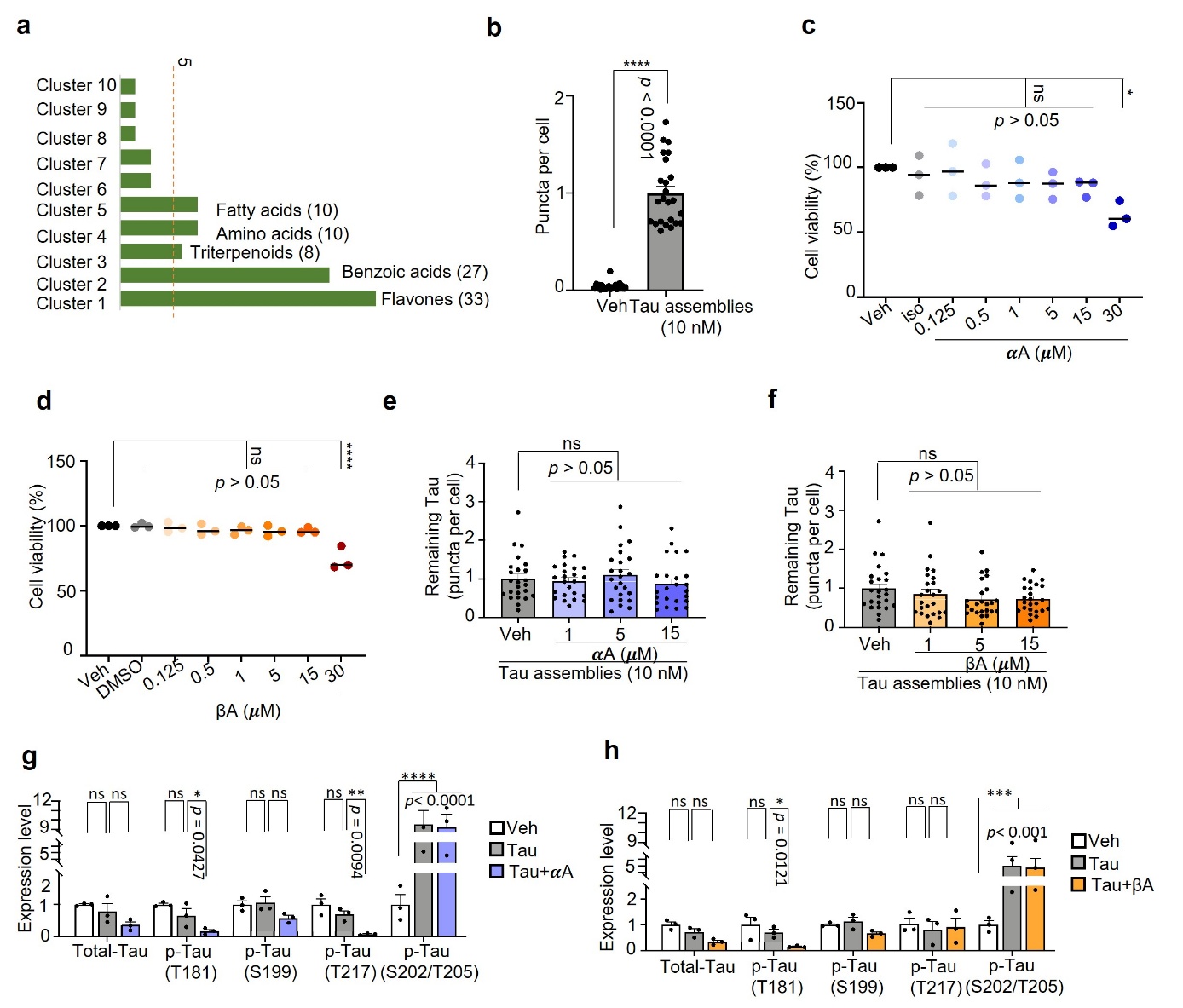


**Supplementary Figure 1. Computational clusters fruit extract-derived neuroprotective compounds and characterization of selected drug candidates α/β-Amyrin in cells.**

**a**, Candidate compounds were assigned to 10 computationally defined clusters as indicated. The number of compounds per cluster (n) is shown for Clusters 1 to 5.

**b**, HEK293 Tau-Venus cells were incubated in the presence of vehicle or heparin-assembled P301S Tau assemblies (Tau seeds) (10 nM, 72h). Aggregation of endogenous P301S Tau-Venus was quantified by counting puncta per cell.

**c**-**d**, Viability measured by MTT assay of HEK294 Tau-Venus cells in the presence of vehicle, αA (0.125 to 30 µM) (c) or βA (0.125 to 30 µM) (d).

**e**-**f**, As in Fig. 2 d-e, concentration dependence of αA (1, 5, 15 µM, 24h) (e) and βA (1, 5, 15 µM, 24h)

**g**-**h**, Bar graph representation of Western blot data is shown in Fig 2f (g, αA and h, βA).

Unless specified elsewhere, data are mean ± S.E.M. Statistical analyses used were as follows: unpaired t-test (b); one-way ANOVA followed by Dunnett's multiple comparisons test (c-f). two-way ANOVA followed by Turkey‘s multiple-comparisons test (g, h) All panels: *n.s*., not significant, **p* < 0.05, ***p* < 0.01, ****p* < 0.001, *****p* < 0.0001.


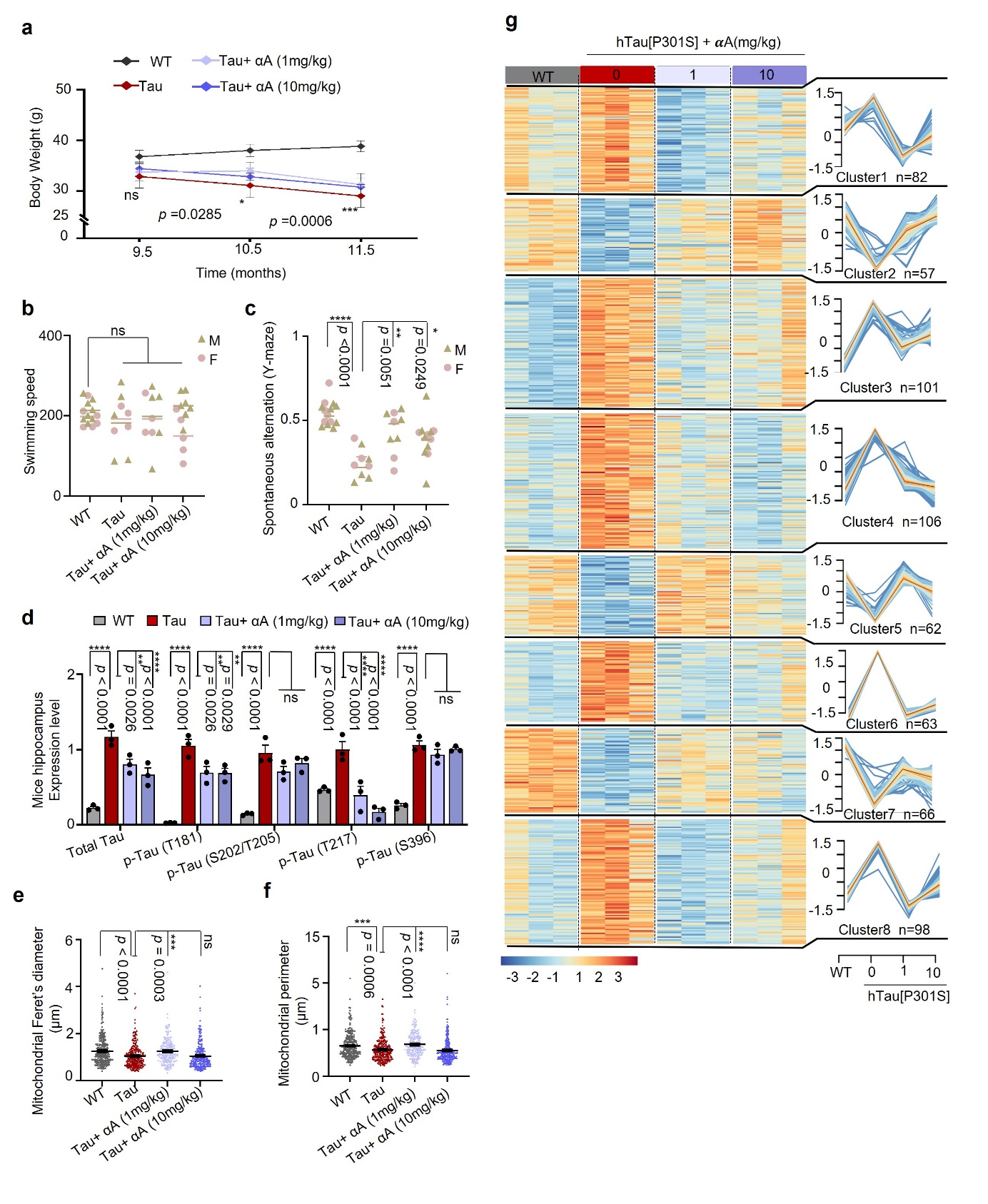


**Supplementary Figure 2. Effects of α-Amyrin on cognitive tests, mitochondrial parameters and normalizes gene expression in hTau[P301S] mice**

**a,** Body weight of WT vs hTau[P301S] mice (n=9-14 mixed gender) treated with vehicle or αA (1, 10 mg/kg) was recorded at 9.5, 10.5 and 11.5 months.

**b,** Swimming speed for WT and hTau[P301S] mice treated with vehicle or αA (1, 10 mg/kg). Mean ± S.E.M. for 9 to 14 mixed gender mice.

**c,** Results of Y maze spontaneous alternation performance test for WT and hTau[P301S] mice in the absence (vehicle) or presence of αA (1, 10 mg/kg).

**d**, Quantifying total Tau and phosphorylated Tau variants in Fig. 4g.

**e**,**f**, Effect of αA (1, 10 mg/kg) on mitochondrial Feret’s diameter (f), and mitochondrial perimeter (g) in hippocampal area of hTau[P301S] AD-like mice (n=3 per group). WT mice were positive control. Data are mean ±S.E.M. of 27 images.

**g**, Heap map showing DEGs in hippocampal tissue from WT and hTau[P301S] mice treated with vehicle or αA (1 or 10 mg/kg) (left), and stratified by gene cluster (right).

Statistical analyses used were as follows: two-way ANOVA followed by Tukey’s multiple comparisons test (a-f); All panels: *n.s*., not significant, **p* < 0.05, ***p* < 0.01, ****p* < 0.001, *****p* < 0.0001.


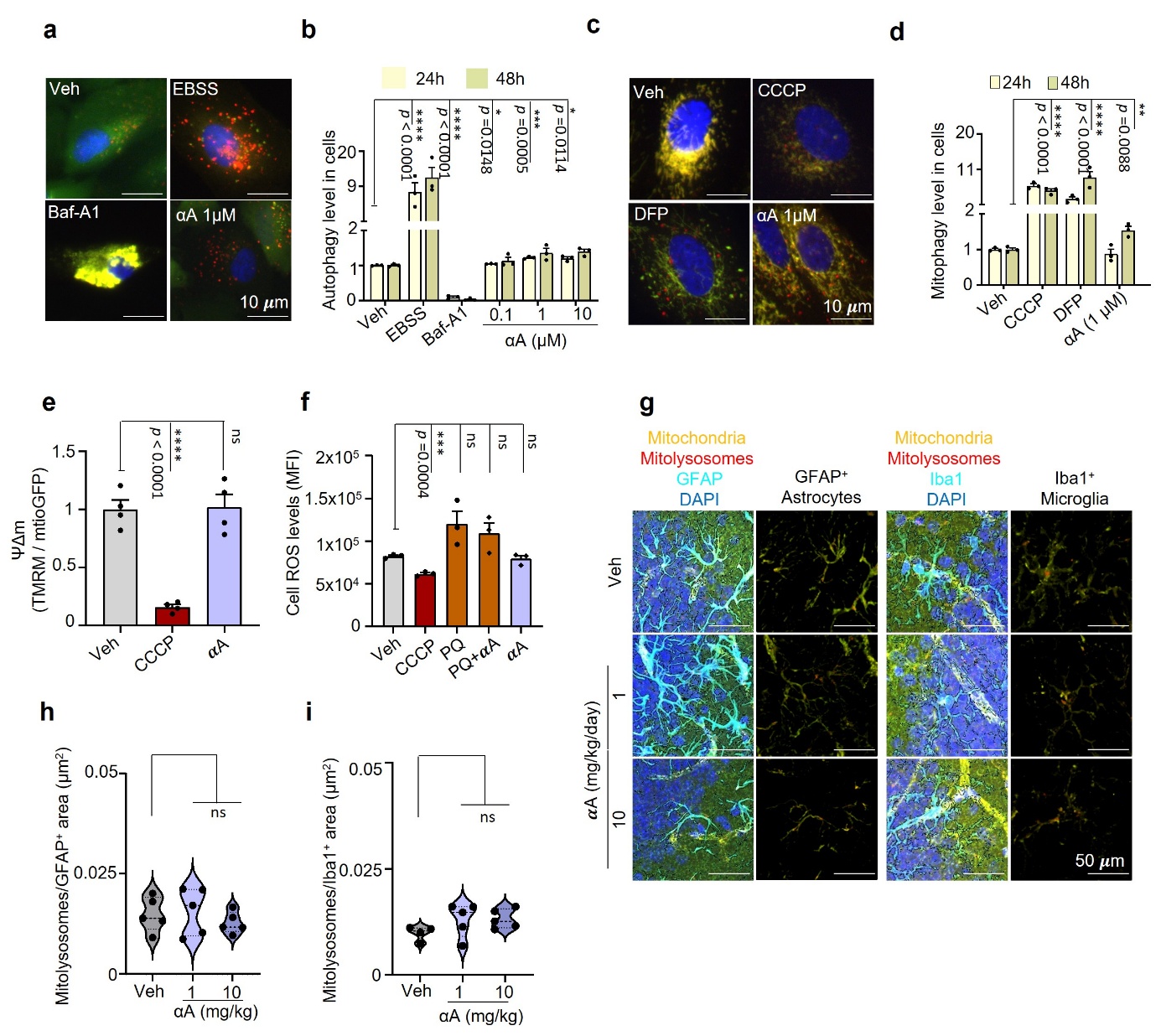


**Supplementary Figure 3. α-Amyrin induces autophagy/mitophagy in cross-species models**

**a**, **b**, Representative images of ARPE-19 cells stably expressing GFP-mCherry-MAP1LC3B were treated with vehicle or αA (1, 10 µM) for 24 or 48 hours (a). Autophagy/Mitophagy were quantified by ratio of fluorescence signal, as described (b). Starvation (EBSS) and Bafilomycin A1 (50 nM) were used as positive and negative controls, respectively. Nuclei were counterstained with DAPI. Scale bar= 10 μm.

**c**, **d**, Representative images of ARPE-19 cells stably expressing mito-QC mitophagy reporter (GFP-mCherry-FIS1101-152) (c) with quantification in (d). Cells were treated with αA (0, 1, 10 µM) as indicated. Mitophagy was assessed 24 and 48 h after treatment. Addition of CCCP (25 µM) selectively triggers PINK1/Parkin-dependent mitophagy, while addition of DFP (1 mM) selectively triggers PINK1/Parkin-independent mitophagy, and were used as positive controls. Mitochondria fluoresce yellow (GFP+ve; mCherry+ve) and mitolysosomes can be identified as red puncta (GFP-ve; mCherry+ve), nuclei were counterstained with DAPI. Scale bar= 10 μm.

**e**, Mitochondrial membrane potential (ΨΔm) was measured using TMRM+mitoGFP in ARPE-19 cells treated with vehicle or αA, as indicated. CCCP were used as controls.

**f**, Cellular ROS were quantified in ARPE-19 cells treated with vehicle or αA, as indicated. CCCP and paraquat (PQ) were used as controls.

**g**, Representative images of brain cryosections of C57BL6/J mito-QC reporter mice injected daily for two days with 1 or 10 mg/kg αA or vehicle. Cryosections were immunostained for cell type-specific markers, astrocytes (GFAP), microglia (Iba1). Nuclei were counterstained with DAPI. Scale bar= 50 µm.

**h**, **i**, Quantitation of effects of αA (1, 10 mg/kg) on mitophagy in astrycotes (h) and microglia (i). ns, not significant.

Mean ± S.E.M of 3 technical and 2 biological replicates is shown. Statistical analyses used were as follows: Two-way ANOVA followed by Šídák's multiple comparisons test (b, d); one-way ANOVA followed by Turkey‘s multiple-comparisons test (e, f); one-way ANOVA followed by Dunnett‘s multiple-comparisons test (h, i) All panels: *n.s*., not significant, **p* < 0.05, ***p* < 0.01, ****p* < 0.001, *****p* < 0.0001.


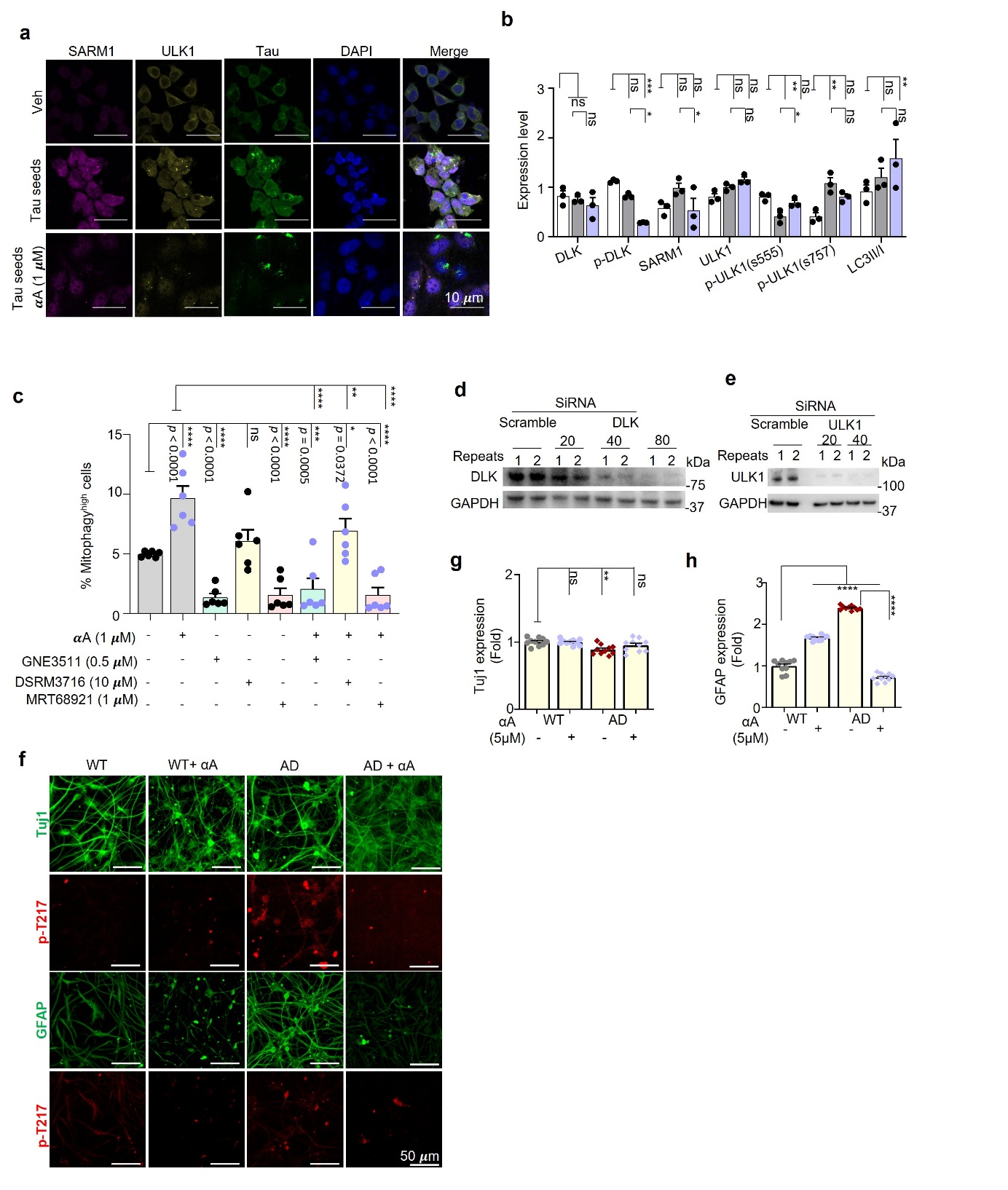


**Supplementary Figure 4. Effect of α-Amyrin on thermal stability of actin and Tau-induced SARM1-ULK1 complex formation as well as co-cultured human microfluidic platform**

**a**, Representative image of co-localization of SARM1 and ULK1 in HEK293T Venus cell in present and absent of Tau seeds and αA (1 µM). Scale bar= 10 µm.

**b**, Quantifying target protein expression in Figure 6h.

**c**, Mitophagy detection in ARPE-19 mito-QC cells in the presence or absence of DLK inhibitor GNE3511, SARM1 inhibitor DSRM3716, ULK1 inhibitor MRT68921 treatment with αA (1 µM, 48h).

**d**, **e**, Western blot quantifying knocks down efficiency in HEK293 Tau-venus cells treated with SiRNA targeting DLK (d), and ULK1 (e). Two biological repeats are shown.

**f-h**, Co-cultured WT and AD microfluidic platform were treated with vehicle or αA (5 µM) and immunostained for neurons (Tuj1), astrocytes (GFAP), and p-Tau Thr217 (**f**). Bar graph shown in fold changes of neurons (**g**), astrocytes (**h**), respectively. Scale bar = 50 μm.

Unless specified elsewhere, data are mean ± S.E.M. Statistical analyses used were as follows: two-way ANOVA followed by Turkey‘s multiple-comparisons test (b,c,g,h)
